# Development of a High-Throughput UHPLC-DMS-MS/MS Method for Targeted Quantitation of Pertinent Phospholipid Classes in Colon Cancer

**DOI:** 10.3390/molecules31030438

**Published:** 2026-01-27

**Authors:** Miriam Wimmer, Olivia I. Coleman, Adam Sorbie, Dirk Haller, Veronika Somoza, Andreas Dunkel

**Affiliations:** 1 TUM Graduate School, School of Life Sciences, Technical University of Munich, Alte Akademie 8, 85354 Freising, Germany; miriam.wimmer@web.de; 2 Leibniz-Institute for Food Systems Biology at the Technical University of Munich, Lise-Meitner-Straße 34, 85354 Freising, Germany; veronika.somoza@univie.ac.at; 3 Chair of Nutrition and Immunology, School of Life Sciences, Technical University of Munich, Gregor-Mendel-Str. 2, 85354 Freising, Germany; olivia.coleman@tum.de (O.I.C.); dirk.haller@tum.de (D.H.); 4 ZIEL–Institute for Food and Health, Technical University of Munich, Weihenstephaner Berg 1, 85354 Freising, Germany; adam.sorbie@med.uni-muenchen.de; 5 Institute of Physiological Chemistry, Faculty of Chemistry, University of Vienna, Josef-Holaubek-Platz 2, 1090 Vienna, Austria

**Keywords:** LC-DMS-MS/MS, phospholipid, rapid work-up, colon cancer

## Abstract

Phospholipids are essential membrane constituents that regulate diverse cellular processes, yet most current workflows rely on relative quantification using high-resolution LC–MS. We developed and validated a highly selective targeted method that couples liquid chromatography with differential mobility spectrometry and tandem mass spectrometry (LC–DMS–MS/MS), providing enhanced selectivity and reduced background noise. The assay quantifies 63 phospholipid species across four classes, achieving excellent recoveries and limits of quantification in the low ng per mg tissue range. Applied to tissues from a colon cancer study in mice, the method enabled the absolute quantification of 47 species, 22 of which were significantly increased in tumor tissue versus adjacent non-tumor tissue. While phosphatidylcholines were the most abundant class overall, the largest fold changes were observed in long-chain phosphatidylglycerol and phosphatidylethanolamine species. LC–DMS–MS/MS thus offers a robust, selective platform for absolute phospholipid quantification and for detecting disease-associated lipid remodeling.

## 1. Introduction

**Figure 1:**
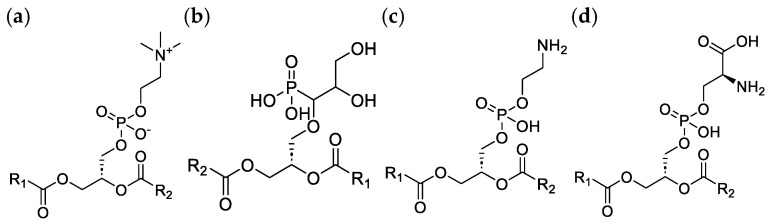
Structural illustration of the composition of applied phospholipids with variable chain lengths (R1/R2 = sn-1 and sn-2 acyl chains): (**a**) phosphatidylcholine (PC), (**b**) phosphatidylglycerol (PG), (**c**) phosphatidylethanolamine (PE), (**d**) phosphatidylserine (PS).

Phospholipids are essential amphipathic constituents of biological membranes. Their structural diversity—including headgroup chemistry and acyl chain composition—governs membrane organization, dynamics, and signal transduction. Variation in headgroups defines the major membrane lipid classes, a cornerstone of comprehensive lipid profiling (Figure 1).

Early phospholipid quantitation relied on thin-layer chromatography (TLC), gas chromatography (GC), fast atom bombardment mass spectrometry (FAB-MS), and solid-phase extraction (SPE) coupled to GC–MS [1,2,3]. With the advent of lipidomics, liquid chromatography–mass spectrometry (LC–MS) has become standard for targeted and untargeted analyses [4,5,6,7,8]. Methodological advances have reduced sample and labor requirements and increased throughput; however, the exhaustive characterization of structurally diverse lipids across complex matrices remains challenging [9].

**Figure 2:**
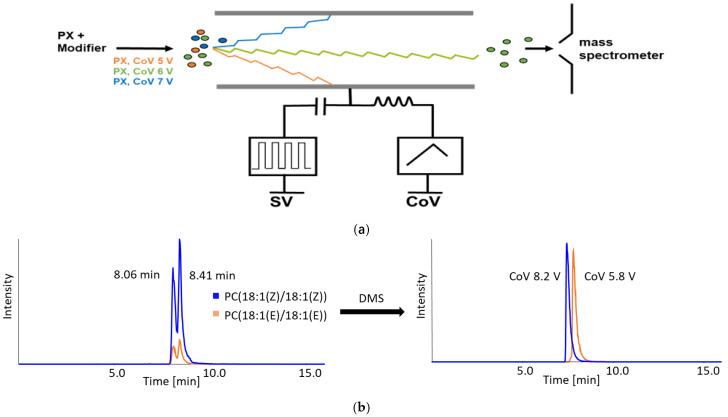
Principle and result of DMS separation in targeted LC-MS/MS analysis. (**a**) Influence of pulse-like frequencies of the compensation voltage (CoV) on analyte modifier clusters at a constant separation voltage (SV). (**b**) LC-MS/MS analysis of isobaric analytes (PC(18:1(Z)/18:1(Z)) and PC 18:1(E)/18:1(E)) without and with application of differential mobility spectrometry (DMS) separation at optimized CoV.

Here, we optimized both sample processing and the analytical workflow to shorten runtime while maximizing analyte coverage in order to elucidate structural differences in phospholipids specific to malignant and normal tissues. To resolve isobaric lipids in targeted assays, we augmented LC–MS/MS with differential ion mobility spectrometry (DMS). DMS provides an orthogonal separation dimension beyond *m*/*z* and fragmentation. Conventional targeted LC–MS often fails to distinguish analytes with identical molecular masses and MS/MS spectra. In DMS, ions traverse a gap between parallel planar electrodes under controlled temperature, carrier gas, and applied asymmetric separation voltage (SV) conditions (Figure 2). Only ions whose trajectories are corrected by an ion-specific compensation voltage (CoV) reach the mass analyzer, thereby excluding unwanted ions. CoV values differ among isobaric and isomeric compounds, and separation can be further tuned with chemical modifiers that influence ion trajectories via transient clustering in protic or aprotic environments.

Interest in phospholipids in tumor biology has grown substantially. Numerous studies report marked differences in phospholipid composition and metabolism between malignant and normal tissues [10,11], with these alterations linked to tumor progression, metastasis, and cancer cell survival. Specifically, key membrane lipids such as phosphatidylcholines (PCs) and phosphatidylethanolamines (PEs) intersect with oncogenic signaling pathways that regulate proliferation and survival [12] and may also shape the tumor microenvironment by modulating inflammatory and angiogenic processes [13]. As structure–function relationships are so far unknown, defining lipid species specific to tumor tissues holds the potential to reveal metabolic adaptations that support cancer cell behavior and tumor–host interactions.

As an initial step, using human plasma, Tuulia et al. successfully analyzed targets across multiple phospholipid classes, including lyso species and variants with diverse acyl chain lengths [14]. Building on this, our implementation of DMS increased sensitivity by suppressing background noise and expanded analyte coverage by resolving isomeric lipids.

We developed and fully validated a high-throughput work-up protocol in combination with an ultra-high-performance liquid chromatography–differential ion mobility spectrometry–tandem mass spectrometry (UHPLC–DMS–MS/MS) method for tissues from a mouse colon cancer study, absolutely quantifying 63 phospholipids across four classes. Enhanced chemical noise suppression and improved selectivity enabled reliable quantification at low concentrations. We applied the method to corroborate previously observed differences among intestinal tissue phenotypes in a mouse cohort and to quantify lipidomic alterations between healthy and tumor tissues [15].

## 2. Results and Discussion

### 2.1. Method Development

LC–MS is widely used to analyze phospholipids across diverse biological matrices [4,5,6,7,8]. While many studies target a limited subset of lipid classes, the comprehensive analysis of isomers and low-abundance species remains challenging. Isomeric compounds are particularly difficult to resolve because they share identical or near-identical masses and often yield similar MS/MS fragmentation patterns. Differential ion mobility spectrometry offers an effective orthogonal separation, since each analyte exhibits a characteristic CoV at a given separation voltage (SV). Applying the CoV facilitates the separation of isomeric and isobaric species and suppresses background noise, thereby improving sensitivity.

All isobaric analytes examined in this study—including PC(18:1(11Z)/18:1(11Z)), PC(18:1(11E)/18:1(11E)), PC(16:0/18:0), PC(18:0/16:0), PE(18:1(11Z)/18:1(11Z)), PE(18:1(9Z)/16:0), PE(18:0/18:1(9Z)), PE(18:1(9Z)/18:0), PG(18:1(11Z)/18:1(11Z)), and PG(18:1(11E)/18:1(11E))—could not be resolved solely by LC interactions with the stationary phase. The UHPLC system employed an aqueous mobile phase containing 5 mM ammonium acetate and an organic phase of acetonitrile/isopropanol containing 5 mM ammonium acetate, adjusted to pH 2.4 with formic acid. Additional resolution of isobaric species was achieved by optimizing and applying characteristic, stepped CoV values. To mitigate oxidative degradation, butylated hydroxytoluene (BHT, 0.005% *w*/*w*) was added to standards and sample extracts.

**Figure 3:**
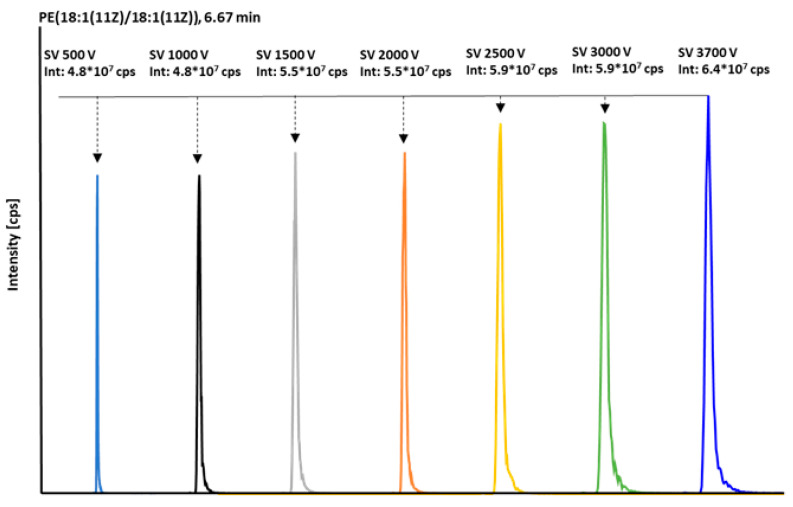
Comparison of the intensities in [cps] of PE(18:1(11Z)/18:1(11Z)) with increasing separation voltage of 500–3700 V at constant retention time (6.67 min).

MS/MS parameters were first optimized in positive electrospray mode (ESI^+^), followed by the refinement of transition-specific settings for each compound. A continuously increasing SV was used for initial ion selection into the DMS cell; the asymmetric field deflects ion trajectories, restricting entry into the subsequent ion path. A compound-specific CoV then counteracts this deflection, steering selected ions into the mass spectrometer. Because the DMS gas-phase equilibrium depends strongly on solvent composition, temperature, and LC flow, analytes were pre-separated chromatographically prior to DMS. CoV values for individual compounds were determined by scanning 0–30 V. For isobaric species, a larger CoV difference was prioritized over absolute signal intensity. The primary optimization parameter was SV, evaluated from 500 to 3700 V in 500 V increments (Figure 3).

Once the optimal SV was identified, it was maintained at this level for all subsequent measurements. For example, PE(18:1(11Z)/18:1(11Z)) exhibited the maximal response at SV = 3700 V, which was used in subsequent experiments. CoV was then fine-tuned to each analyte, considering retention behavior and ionization efficiency. Most compounds were resolved using a 0–30 V CoV scan in 1 V steps; isobaric pairs required finer resolution. SV influenced ion trajectories within the DMS cell, whereas CoV governed the stability of analyte–modifier clusters, enabling the transmission of the selected ions to the mass spectrometer (Figure 2a). Compound-specific CoV settings allowed the separate analysis of isomeric phospholipids within a single run. Differences in transient clustering between cis and trans isomers supported their separation and quantification. Notably, small CoV adjustments could significantly enhance target signal intensity while suppressing interferences.

**Figure 4:**
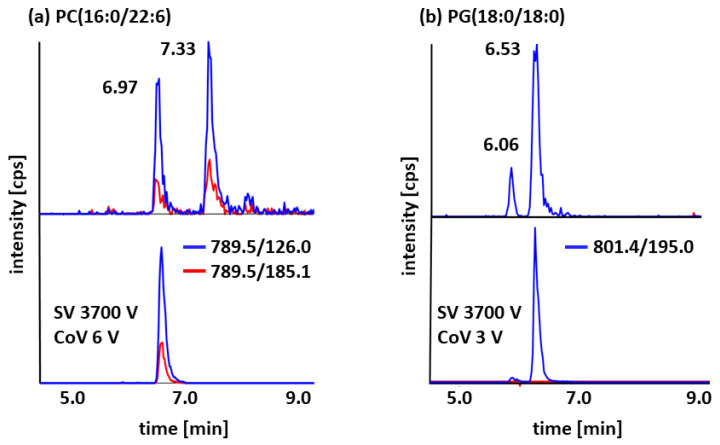
Sections of representative LC-MS/MS chromatograms demonstrating the selectivity improvement of DMS application. (**a**) Extracted ion chromatograms of PC(16:0/22:6) analyzed by conventional LC-MS/MS (top) and using LC-DMS-MS/MS with optimized parameters (bottom). (**b**) Extracted ion chromatograms of PG(18:0/18:0) using LC-MS/MS (top) and LC-DMS-MS/MS (bottom).

**Figure 5:**
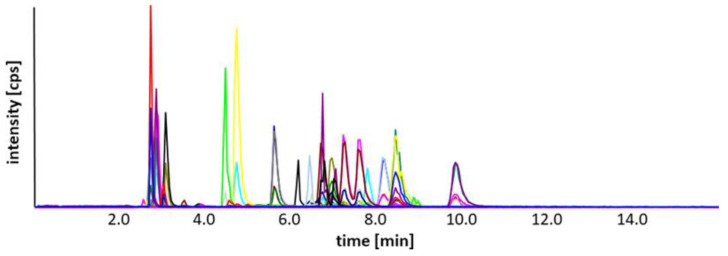
Optimized UHPLC-DMS-MS/MS chromatogram of 63 phospholipids included in the method. The LC-DMS-MS/MS conditions refer to isopropanol as modifier, SV 3700 V, and optimized CoV values for each analyte.

The selective assignment of signals to target analytes and concurrent reduction in potential false-positive signals enabled comprehensive profiling with unambiguous compound qualification (Figure 4a,b). Due to the reduction in background noise, signal-to-noise ratios were improved as well, providing, in parallel, a clear reduction in lower detection and quantitation limits supporting the quantitative analysis of trace analytes under optimized DMS conditions. The final DMS settings applied for subsequent measurements and method validation are reported in Table S3. With the exception of PC(16:0/18:1), all isobaric pairs were resolved from their corresponding isomers. Since PC(16:0/18:1) and its positional isomer PC(16:0/18:1) showed no sufficient separation in the DMS, independent of applied parameters and modifier selection, a separate detection was not achieved, and therefore, results are reported as the sum of the isomeric forms (Figure 5).

For method work-up validation, sample preparation included a two-hour equilibration of the internal standard solution within the tissue prior to extraction and filtration, without further dilution. This equilibration step allows internal standards to diffuse into the matrix and mimic analyte behavior. BHT (0.005% *w*/*w*) was included in all samples, standards, and dilutions throughout the workflow.

### 2.2. Method Validation

A major challenge in validation is the sample matrix: a truly analyte-free (blank) intestinal matrix is not available. Consequently, method validation for membrane lipids was performed in native (non-chemically purified) porcine intestinal tissue. To isolate the performance of individual validation steps, endogenous analyte concentrations in the tissue were measured and subtracted as blank values.

Limits of detection (LoDs) and quantification (LoQs) were established in-matrix by serially diluting spiked porcine intestinal tissue to the detection limit, with selectivity and linearity summarized in Table S4. LoDs and LoQs were calculated using signal-to-noise (S/N) criteria following Brunetti [16]: LoD at S/N = 3.0 (≈99% detection probability) and LoQ at S/N = 10.0, based on the lowest concentration meeting the specified ratio. To mitigate oxidation, BHT (0.005% *w*/*w*) was included throughout.

Across all phospholipid classes, LoDs spanned broadly from 0.02 ng/mg for LPG(14:0/0:0) to 860.76 ng/mg for PS(18.0/18.1). Notably higher LoD values were observed for a subset of four PCs, ranging from 2653.26 ng/mg for PC(18:1/18:0) to 5990.03 ng/mg for PC(18:0/22:6), which could be associated with a relatively high background signal not removed by the DMS. Within classes, lyso-phospholipids generally exhibited lower LoQs than their corresponding diacyl species. No consistent effect of acyl chain length on LoD/LoQ was observed. Median values and range of LoD and LoQ are provided as summary statistics in Table 1 and illustrate comparable dynamic ranges across analyte classes.

To ascertain the lowest quantification content, it is necessary that the calibration data of the analyte be linear. The applicability of the linear regression calculation requires a linear relationship between the substance content and the measured value. In the event of the Mandel adjustment test being utilized, a significant superiority of the linear calibration function (1) or the 2nd degree calibration function (quadratic calibration function, 2) must be present [17]. The residual standard deviation of both functions can then be used to calculate a test value, which can be compared with the corresponding F value.

y = ax + b(1)

y = ax^2^ + bx + c (2)

Mandel’s F-value is a sum-of-squares F-test used to check calibration linearity by comparing a straight-line fit with a quadratic fit to the same data. It represents the ratio of the reduction in residual sum of squares gained by adding the quadratic term to the residual mean square of the quadratic model; under the null hypothesis of linearity, it follows an F distribution with 1 and n − 3 degrees of freedom. A large F (small *p*-value) indicates significant curvature (lack of linearity); a small F suggests the linear model is adequate. The results of the adaptation test according to Mandel and a visual analysis confirm the linearity of the calibration line (Table S4) in the form of the coefficient of determination R^2^. The values presented in this table are the result of a double determination and, thus, can be accompanied by their respective standard deviations.

Coefficients of determination (R^2^) were predominantly ≥0.98 (Table S4), confirming the linear response over the working range. Four analytes—PS(14:0/14:0), PS(16:0/18:1), PS(18:0/18:1), and PC(18:1(Z)/18:1(Z))—showed slightly lower linearity (R^2^ = 0.97), attributable to low-level endogenous background in the non-blank matrix. All values represent duplicates and are reported with their standard deviations.

**Figure 6:**
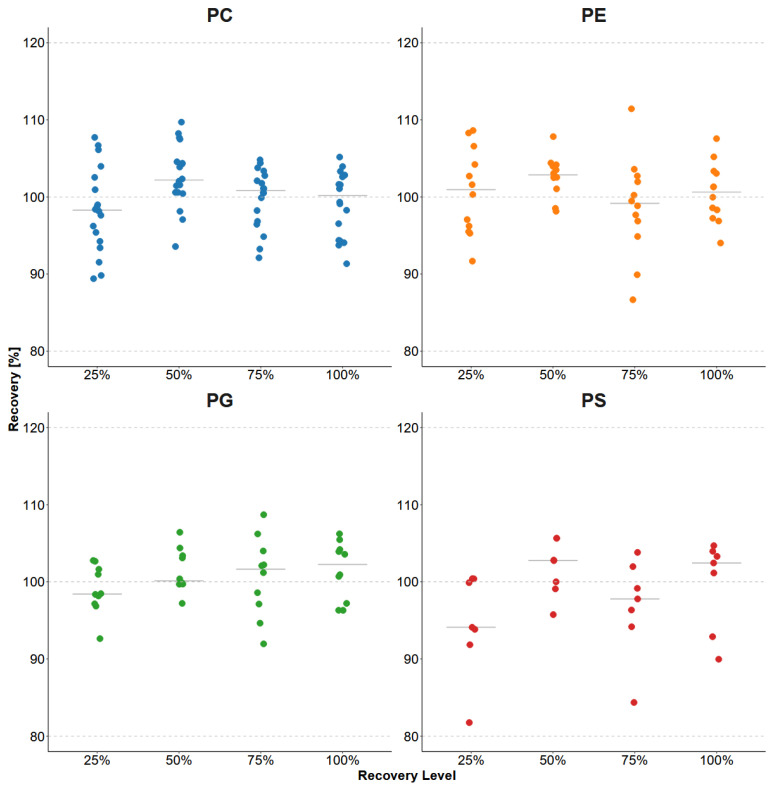
Summary of the percentage recovery results of analytes in pig intestine tissue across spiking levels (25%, 50%, 75% and 100%) grouped by phospholipid classes PC (*n* = 19), PG (*n* = 11) PE (*n* = 11), and PS (*n* = 7). Data points represent individual analytes at a specific spiking level, and horizontal bars indicate the distribution median.

Recovery rates were determined by selecting four concentration ranges based on the concentrations of the analytes to be detected in the tumor. The lowest spiking level (25% recovery level) of the mean working range was individually adjusted to all analytes up to the working range of the tumor tissue itself (recovery level 100%) in 25% steps. Spiking was performed prior to tissue processing, and recoveries were determined by LC–DMS–MS/MS. An acceptance range of 80–120% was predefined; all analytes met this criterion. The lowest recovery was 81.77% for PS(16:0/16:0), and the highest was 111.44% for PE(14:0/14:0) (Figure 6; full data in Table S5). The comparison of the recovery levels across the four phospholipid classes showed only marginal differences and no systematic concentration-dependent shifts. Percent relative standard deviations (%RSDs) across all ranges of spiking levels were 1.76–17.84, with PS(16:0/18:1) showing the largest %RSD values (15.12% and 17.84%).

Precision was assessed as intraday (three independently extracted replicates measured repeatedly within 24 h) and interday (measurements on three separate days). Intraday measurements encompass the repeated assessment of three samples of pig intestine, extracted in equal proportion, with the incorporation of internal standards and a spiking concentration of PCs of 2.08 µmol/L (PC(14:0/14:0))–403.76 µmol/L (PC(16:0/18:1)/PC(18:1/16:0)), PEs of 0.87 µmol/L (PE(14:0/14:0))–143.75 µmol/L (LPE(18:0/0:0)), and PGs of 0.89 µmol/L (PG(14:0/14:0))–135.03 µmol/L (PG(18:0/22:6)), corresponding to tenfold tumor concentrations. Acceptance was defined as a coefficient of variation (CV) not exceeding a value of 15%. CVs were generally <6% for both intra- and interday measurements (Table S6). Modestly higher CVs were observed for PCs in intraday tests and for PEs in both intra- and interday tests, yet all remained within acceptance limits.

Enhanced chromatographic peak shape and ion mobility resolution enabled the clear separation of isobaric species, while future studies will investigate adduct formation involving the ammonium acetate in the mobile phase more in depth. Using LC–DMS–MS/MS, we consolidated quantification across four structurally distinct phospholipid classes into a single, validated assay with expanded analyte coverage.

Comparative targeted quantitation methods using triple-quadrupole UHPLC–MS in the literature rarely include isobaric phospholipid species with individually analyzed configurational isomers and always express their sensitivity on a per volume basis, while the current study provides additional selectivity and possesses validation information per tissue weight, including sample work-up. A UHPLC–MS/MS method spanning ten phospholipid classes reported class-specific LoDs from 0.04 to 33 pmol/L (matrix-matched), with corresponding LoQs of 0.1–110 pmol/mL; the broad range reflects class-dependent ionization and background [18]. A scheduled multiple reaction monitoring workflow using class-matched isotopic standards determined LoDs for representative classes in human plasma of 0.245 pmol/L for PE, 0.291 pmol/L for PG, 13.0 pmol/L for PC, and 41.9 pmol/L for PS and PA, i.e., femtomolar to low-picomolar levels on the concentration scale used [19]. In contrast to the present study, these methods were developed using simulated fragment spectra from lipidomics libraries and only performed relative targeted quantitation of the target analytes. This resulted in a significantly higher number of included structures, since the coverage of our method is primarily limited by the commercial availability of pure reference compounds. Focused assays for specific subclasses showed even higher sensitivity: a lysophospholipid UHPLC–MS/MS method achieved LoD/LoQ of 0.2/0.8 nM in serum for LysoPC/LysoPE, and an oxidized phosphatidylcholine method reached 0.01 pmol (10 fmol) on-column for PC-hydroperoxides [20]. Nevertheless, sensitivity is in a similar range to the present method, while this approach would require multiple specific methods to achieve an equivalent analyte coverage, resulting in longer analysis times and limiting its applicability to small sample amounts.

Beyond the application of highly sensitive MS instrumentation, additional levers can demonstrably shift LoDs. First, nanoscale or microflow LC enhances ESI efficiency: studies comparing nanoLC to conventional flows documented 10–1000× intensity gains and on-column LoDs down to a few femtomoles for PCs, albeit with higher operational complexity than standard UHPLC [21]. Second, sample preparation influences background and recovery. Methyl tert-butyl ether (MTBE) extraction is widely adopted for broad lipid coverage with high recoveries and minimal chlorinated solvent use, while comparisons across extraction systems show that minor but analytically important classes such as phosphatidylinositols (PIs) and lysophospholipids (LPLs) are most sensitive to the solvent system. Oxidation-prone analytes require rapid processing at low temperature, as in the present study, with antioxidants to ensure that results reflect biology rather than artifacts [22,23].

In summary, the presented method, incorporating differential ion mobility, provides a unique combination of high-throughput work-up for small tissue samples in the low mg range and highly selective absolute quantitation, with full matrix validation of the complete workflow, including recovery estimation at the tissue level.

### 2.3. Application of the Optimized Method to Non-Tumor Tissue and Colorectal Cancer (CRC) Tissue in Mice

Colorectal cancer (CRC) has been shown to modulate lipid metabolism, with phospholipids at the center of this transformation. As the dominant structural and signaling lipids of cellular membranes, phospholipids govern membrane architecture, receptor organization, vesicle trafficking, and stress responses. Despite growing evidence, quantitative, isomer-resolved measurements in tissue are limited by isobaric complexity and matrix effects. This motivates the application of robust analytical strategies for absolute phospholipid quantification to develop molecular models of CRC-associated remodeling and uncover biomarker and therapeutic opportunities [15,24].

**Figure 7:**
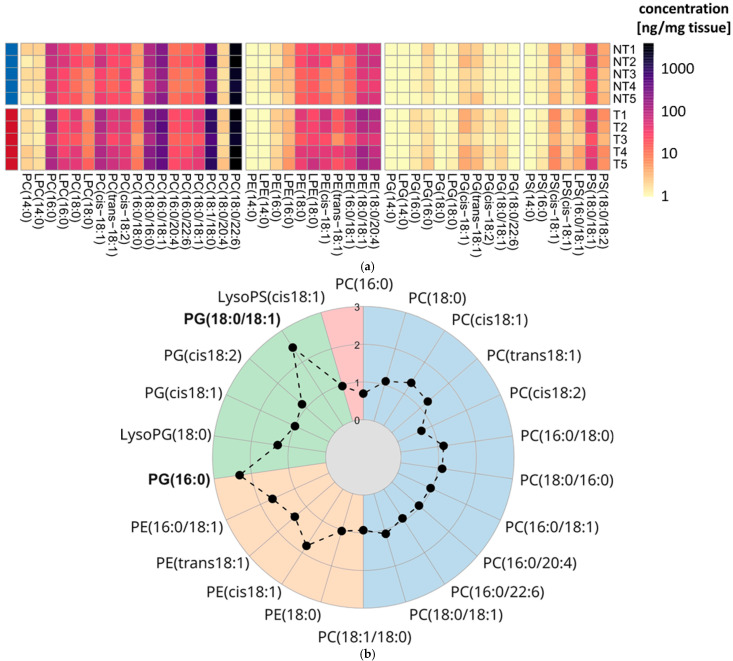
Visualization of quantitative results from the optimized LC-DMS-MS/MS applied to non-tumor tissue and CRC tissue in mice. (**a**) Heat map of absolute concentrations [ng/mg tissue] of all phospholipid classes in tumor samples (T) compared to absolute concentrations ([ng/mg]; NT) of tumor-free samples. Sample groups are indicated by the color bar (left border) indicating NT (blue, *n* = 5) and T (red, *n* = 5) samples (**b**) Radar plot of log_2_-fold changes between T and NT samples for phospholipid features with a significant mean concentration difference between phenotypes. Features possessing a log2-fold change > 2 are highlighted in bold.

To interrogate these changes, we quantified absolute concentrations across four phospholipid classes in tumor (T) and adjacent non-tumor (NT) tissues from mouse cancer models using LC–DMS–MS/MS. Animals were maintained under standardized housing and diet to minimize environmental confounding, enabling robust comparisons of molecular lipid profiles between phenotypes. Quantitative data were obtained for 47 targeted phospholipid species and are summarized as a heatmap in Figure 7a (full data given in Table S7). Concentrations differed markedly across classes: PCs were most abundant, with selected long-chain species—PC(18:0/22:6), PC(18:0/18:1), PC(16:0/18:1), and PC(18:0/16:1)—reaching up to 3730 ng/mg tissue. PEs showed the next-highest levels, up to 158 ng/mg for PE(18:0/18:1). Among PSs, only PS(18:0/18:1) exceeded 6.5 ng/mg. PGs were lowest overall, rarely exceeding 1 ng/mg, observed only in a few samples for cis- and trans-PG(18:1). Within each class, species containing at least one ≥C18 acyl chain were consistently more abundant than shorter-chain analogs; conversely, species containing myristate were at least an order of magnitude less abundant than other phospholipids.

Although absolute concentration levels of individual phospholipids in tissues are rarely reported, the results are in line with relative data observed in multiple studies on various types of membranes. In mammalian plasma membranes, PC typically constitutes roughly one third to nearly one half of total phospholipids, with representative ranges of 35–45 mol%, while PE contributes about 15–25 mol% and sphingomyelin (SM) about 10–20 mol%; the principal anionic phospholipids—phosphatidylserine (PS) and phosphatidylinositol (PI)—each account for approximately 5–10 mol%, with phosphoinositides such as phosphatidylinositol 4,5-bisphosphate (PIP2) comprising ≤1 mol% of total phospholipids [24,25,26]. Across tissues with high oxidative capacities, such as the heart and brain, plasmalogens (vinyl ether phospholipids) can constitute large fractions of PE and, to a lesser extent, PC, with PE plasmalogens frequently representing 20–40% of total PEs, a feature with implications for redox buffering and membrane biophysics [27]. Focusing on phospholipid levels in gut tissue, analysis by bulk LC–MS(/MS) and spatial matrix-assisted laser desorption/ionization (MALDI) MS imaging in epithelial biopsies, surgical specimens, and mucus revealed that PC and PE are typically among the dominant membrane phospholipids, while SM, PS, and PI are present at lower relative abundances, although composition varies strongly by membrane domain and gut segment, as shown in classic basolateral and brush-border preparations from the intestine and colon. In colonic epithelial basolateral membranes, for example, PE can be markedly enriched, underscoring how domain-specific lipid composition shapes membrane biophysics and protein function. PCs are also the principal phospholipids in the intestinal mucus barrier—often accounting for the majority of mucus phospholipids—and are supplied to the lumen by epithelial transport that includes a tight-junction-dependent paracellular route and an ileal secretory source feeding distal mucus [28,29,30].

The heatmap delineated differences in the abundances of individual phospholipids. To resolve phenotype-specific variation between tumor and non-tumor colorectal tissue, we performed analyte-level comparisons for each species. Inferential analyses used the nonparametric Wilcoxon rank-sum test because normality could not be assumed given the small sample size. Of the 47 detected analytes, 22 differed significantly between groups (*p* < 0.05), 12 of which were PCs. To further examine phenotype-dependent changes among these analytes, we calculated log_2_ fold changes (log_2_FC) and displayed them in a radar chart (Figure 7b). The gray inner circle denotes the NT group median (log_2_FC = 0), whereas positive values toward the outer sectors indicate higher concentrations in the T group. All 22 significant analytes exhibited positive log_2_FC values; PG(18:0/18:1) and PG(16:0) showed the largest increases and were the only species with log_2_FC > 2. By contrast, fold changes within the PC class—the most abundant analyte group—clustered around a log_2_FC of approximately 1.

These results are mostly in line with literature results, that colorectal cancer (CRC) tumor tissue undergoes extensive remodeling of glycerophospholipids to sustain proliferation, shape signaling, and tune the tumor microenvironment. Recent tissue studies in CRC indicate tumor-biased changes in specific PC and SM species, shifts in ether PE and other classes, and altered lysophospholipids, e.g., increased lysophosphatidylinositols and lysophosphatidylglycerols in some cohorts, reflecting reprogrammed membrane anabolism and signaling. Spatial lipidomics further reveals tumor–stroma compartmentalization; in CRC peritoneal metastases, sulfatides are tumor-associated while stromal regions are enriched for PI(18:0/20:4), highlighting microenvironment-specific phospholipid and sphingolipid distributions that may have a bearing on invasion and therapeutic targeting [31,32].

Using multiple analytical platforms including LC-MS- and MS-based imaging by MALDI or DESI, a consistent shift from energy-storing neutral lipids toward membrane-forming phospholipids and related polar lipids was described. In matched tumor-versus-adjacent mucosa, triacylglycerols fall while total phospholipids, sphingomyelin, and cholesterol rise, supporting increased membrane biogenesis and raft formation in rapidly dividing CRC cells. This reprogramming is also reflected in fatty acyl composition: tumor tissue tends to be enriched in saturated and polyunsaturated acyl chains relative to monounsaturated chains, typically abundant in storage lipids [33].

PCs are the dominant structural phospholipid in colon epithelium and are further enriched and remodeled in CRC. Spatial lipidomics by MALDI-MS imaging demonstrates that PC(16:0/18:1) and its precursors, lysophosphatidylcholines (LPC 16:0 and LPC 18:1), localize to tumor regions and are depleted in nearby normal mucosa. In addition, former membrane biochemistry studies in human CRC biopsies showed that a higher PC/PE ratio correlates with metastatic disease, an observation that interlinks with the modern imaging and enzymology data implicating PC enrichment in aggressive phenotypes [34,35].

PEs are also reprogrammed depending on the compartment and context. Tumor tissue lipidomics frequently demonstrated specific PE species being elevated, particularly longer and more polyunsaturated PEs, suggesting a remodeling continuum along the adenoma–carcinoma sequence. In addition, exosome lipidomics indicates that certain PE species decrease in metastatic versus nonmetastatic contexts, hinting that ether lipid allocation differs between intracellular membranes and secreted vesicles during dissemination [36,37]. PS species are less abundant than PC or PE but are considered functionally consequential. Several studies detected higher PS and ether-linked PS species in tumors with heterogenous results across cancer types and CRC subtypes, while within this study, only lysoPS(cis-18:1) was among the analytes showing a significant upregulation [38,39]. Literature results from lysophosphatidylserines and other phospholipid classes not covered in this study, e.g., lysophosphatidylinositol, highlight their role as mediators in CRC tissue. Targeted LC–MS analyses of resected human tumors have repeatedly found that LPI and LPS are significantly increased in cancer versus matched normal mucosa, with 18:0 species especially prominent, while lysophosphatidic acid is decreased—implying active but rewired lysophospholipid metabolism within tumors. Larger tissue datasets also report that, although total LPL content may rise in tumors, individual LPL classes behave divergently between studies, likely reflecting inter-patient heterogeneity, sample preparation differences (whole tissue versus isolated cells), and the variable activity of enzymes such as autotaxin and the lysophosphatidylcholine acyltransferase family [40].

Beyond phospholipid class totals, imaging and bulk lipidomics consistently highlighted the accumulation of specific PC species with 32:1, 34:1, and 36:1 composition (reflecting palmitate/oleate and stearate/oleate pairs) in tumor tissue, while many PUFA-containing phospholipids not covered in our method are variably decreased or regionally redistributed. Future additions to the presented method could enhance coverage in this area, as PUFAs are well characterized for modulating cancer risk and progression by altering membrane composition, signaling, and inflammation. n-6 PUFAs (e.g., arachidonic acid) generate prostaglandins and leukotrienes that can promote inflammation, proliferation, and angiogenesis, whereas n-3 PUFAs compete with arachidonic acid to produce less inflammatory, pro-resolving mediators.

Together, these data argue that CRC cells both synthesize and import fatty acids to rework the phospholipidome toward species that optimally support membrane order, curvature, and signaling [41,42]. These patterns have been consolidated using clinical correlations and spatial studies. Tumor-compartmentalized phospholipid signatures, e.g., PC(16:0/18:1) and LPC(16:0/18:1), map precisely to malignant glands by MALDI-MSI and can reveal local effects in histologically normal-appearing mucosa adjacent to tumors—implicating phospholipid metabolism in early, localized transformation.

In summary, the application of the UHPLC-DMS-MS/MS method enabled the robust absolute quantitation of 47 phospholipid species across four classes in matched tumor (T) and non-tumor (NT) mouse colorectal tissues, with 22 analytes showing significantly elevated concentrations in T.

## 3. Conclusions

We established and fully validated a targeted LC–DMS–MS/MS workflow for the absolute quantification of phospholipids from low-milligram tissue amounts, addressing key limitations of conventional LC–MS approaches. Differential ion mobility provided orthogonal selectivity that resolved nearly all isobaric/isomeric species, suppressed background noise, and improved sensitivity. In-matrix validation in porcine intestine demonstrated excellent method recovery with limits of detection down to 0.01 ng/mg tissue. Applied to colorectal cancer mouse tissue, the assay quantified 47 species and revealed significant tumor-associated remodeling: 22 analytes were elevated in tumors, with the largest fold changes in long chain phosphatidylglycerol and phosphatidylethanolamine species. This single run, high-selectivity method enables robust, tissue weight-based absolute lipid quantification and sensitively captures disease-related membrane remodeling. Expanding reference standards and coverage to additional classes and matrices will further extend its utility for biomarker discovery and mechanistic studies.

## 4. Materials and Methods

### 4.1. Phospholipids

The deuterated internal phospholipid standard utilized is a mixture (UltimateSPLASH™ ONE (Avanti Polar Lipids, Alabaster, AL, USA)) of 69 deuterated analytes for lipidome analysis and contains 15 phospholipids essential for the developed method (Table S2). Ultimate Splash standard solution and all phospholipids (63) were procured from Avanti Polar Lipids.

### 4.2. Chemicals and Solvents

Acetic acid 99–100% was purchased from Carl Roth (Karlsruhe, Germany) and 5 M ammonium acetate solution, acetonitrile, and isopropanol (both LC-MS grade) obtained from Honeywell (Seelze, Germany). Methanol-d_4_, cholorforme-d_1_, dimethylesulfoxide-d_6_, and 2.6-ditertbutyl-4-methylphenol (BHT) were received from Sigma Aldrich (Steinheim, Germany) and water for UHPLC separation was purified using a Milli-Q water advantage A 10 water system (Millipore, Molsheim, France).

### 4.3. Sample Preparation for Quantitation of Phospholipids

#### 4.3.1. Tumor-Analyte-Free Tissue Extract Preparation

A 2 mL bead beater tube (CK14 2 mL, 1.4 mm inner diameter, Bertin Technologies, Montigny-le-Bretonneux, France) was utilized to weigh 20 mg of tumor-free, purified porcine intestinal tissue filled with ceramic beads. The volume of methanol (0.005% BHT) and the internal isotope-labeled processing standard solution was adjusted to 990 µL and 10 µL, respectively, to compensate for any losses incurred during sample preparation. Subsequently, the homogenization process was conducted at 13,000 rpm (3 × 25 s, 2 × 20 s pauses) using a bead beater (Precellys Evolution Homogenizer, Bertin Technologies) equipped with a Cryolys cooling module (Bertin Technologies, cooled with liquid nitrogen). Following this, the transparent supernate was separated through centrifugation of the suspension (10 min, 13,000 rpm, 0 °C) using an Eppendorf Centrifuge 5415R (Eppendorf, Hamburg, Germany). Thereafter, 99 µL of the filtered supernate was aliquoted with 1 µL UltimateSPLASH™ONE, mixed, and injected into the LC-MS/MS system for targeted analysis. The residual volume of the filtered sample was transferred to cryogenic tubes and stored at −80 °C for subsequent analysis. For each analyte, 1 mg of the substance was diluted with 1 mL of methanol (0.005% BHT) to create standard solutions with concentrations ranging from 0.07 to 3.21 mmol/L for phosphatidylethanolamine. 0.24 and 3.57 mmol/L for phosphatidylcholines, 0.28 and 3.09 mmol/L for phosphoglycerols, and 1.24 and 2.78 mmol/L for phosphatidylserines. The concentration range for phosphocholines was 4–3.09 mmol/L, for phosphatidylglycerols it was 0.28–3.57 mmol/L, and for phosphatidylserines it was 1.24–2.78 mmol/L. For the purpose of method development, a 1/10 dilution (methanol, 0.005% butylated hydroxytoluene) was employed.

#### 4.3.2. Preparation of Stock Solutions and Calibration Curves

UltimateSPLASH™ ONE was utilized in accordance with the stipulated guidelines, employing the recommended concentrations of deuterated phospholipids (31.78 mmol/L and 192.65 mmol/L) and storing it at −80 °C until it was needed. Deuterated working standards were prepared in solution with methanol (0.005% BHT) to a working concentration of 11.48 mmol/L for C16:0-d_3_ and 3.65 mmol/L for C18:0-d_4_. For the calibration curves, the stock solutions of all phospholipid classes were combined in individual concentrations and diluted in several 1:1 dilution steps with methanol (0.005% BHT), aliquoted, and mixed with isotope-labeled work-up solution and 1 µL UltimateSPLASH™ ONE to achieve concentrations of 0.003–50 mmol/L. The concentrations of the analytes were set at 1 mmol/L, while that of the UltimateSPLASH™ ONE mixture was set at 0.31–1.93 mmol/L. Following analysis, calibration curves for all phospholipids were generated by plotting the peak area ratio of analyte to deuterated internal standard against the concentration ratio of analyte to internal standard using linear regression (R > 0.985).

#### 4.3.3. Method Validation

In order to ensure standardized validation, it is necessary to use a comparable or similar matrix without analytes in sufficient quantity in relation to the mouse gut. Limit tests, precision measurements, and recoveries are carried out in several processing steps and should be performed on the same matrix to ensure a high degree of comparability. Since a single mouse intestine does not provide sufficient material for all validation steps, method development is carried out on a matrix similar to that of a mice intestine. However, a matrix completely free of analytes was not available.

In order to ascertain the recovery rate of membrane lipids, porcine intestinal tissue was spiked with four defined, but distinct, concentrations (percentage decreases of 25%, 50%, and 75%) of the individual phospholipids, in addition to a fixed amount of an internal work-up standard. Healthy porcine colon tissue was provided by the Chair of Reproductive Biotechnology, School of Life Sciences, Technical University of Munich (Freising, Germany). Samples were then processed in accordance with the methodology that had been previously described. A control sample was prepared by adding the internal work-up standard to pig intestinal tissue without spiking the analytes. This control sample was then extracted and quantified as recovery samples using UHPLC-DMS-MS/MS.

The limit of detection (LoD) of all phospholipids was defined as the concentration at which the areas of 10 analyte-free matrix blanks, added by a factor of three times the standard deviation of the analyte-free matrix blanks, were smaller than the area of the smallest analyte peaks. The limits of quantification (LoQs) were defined as the concentrations at which the average area of ten analyte-free matrix blanks, summed by ten times the standard deviations of the analyte-free matrix blanks, is smaller than the area of the analyte peak. Lower limits of detection (LoDs) and lower limits of quantification (LoQs) were determined using a 24-point calibration curve, in which an analyte-free matrix extract of extracted tumor-free porcine intestine in methanol (0.005% BHT) was added to individual concentrations for each analyte. Assessment of the calibration curve’s linearity employed the Mandel test, and the results indicated that each phospholipid exhibited linearity—details of the calculation are provided in the Supplementary Materials.

The precision of the measurements was determined by analyzing three purified aliquots of the porcine intestine tissue extract on non-consecutive days for all analytes. The interday precision was expressed as the relative standard deviation of replicate analyses on three days. To ascertain the intraday precision of the method, five replicate analyses were conducted and expressed as the relative standard deviation of the determined area ratio of analyte and deuterated internal standard.

### 4.4. Liquid Chromatography–Differential Ion Mobility–Tandem Mass Spectrometry (LC-DMS-MS/MS)

The used measurement system comprises a liquid chromatography triple-quadrupol/linear ion trap mass spectrometer with differential ion mobility spectrometry (LC-DMS-MS/MS; QTRAP 6500^+^ mass spectrometer with SelexION^+^ DMS cell; Sciex, Darmstadt, Germany) for the detection of fragment mass spectra in multiple reaction monitoring (MRM) mode using positive electrospray ionization (ESI). The mass spectrometer employed a carrier gas of pure nitrogen, which was augmented with various chemical modifiers, including methanol, acetonitrile, and 2-propanol, when utilizing the DMS cell. Optimizations of the method was performed on various parameters, including the chemical addition, its flow rate (low = 363.6 µL/min, high = 738.5 µL/min), separation voltage (SV = 500–3700 V), DR (DMS Resolution Enhancement: open = 0 psi, off = 10 psi, low = 20 psi, medium = 30 psi, high = 40 psi), the offset (DMO = −30–30 V), and the DMS temperature (DT: low = 150 °C, medium = 225 °C, high = 300 °C), as well as the collision gas (CAD = low, medium, high). DMS offset (DMO) can be adjusted between −30 and 30 volts, while the DMS temperature can be set to low (150 °C), medium (225 °C), or high (300 °C). Collision gas (CAD) can be set to low, medium, or high. Finally, the voltage offset (DMO) was evaluated in 1-volt increments. Optimization parameters were listed in the comparison of characteristic parameters, with the compensation voltage (CoV) set at 0.30 V per step. In consideration of the alterations in the values of the CoV, final parameters for quantitative measurements were established as follows: 2-propanol was employed as a chemical modifier in a high flow rate (738.5 µL/min), a high DMS temperature (300 °C), a separation voltage of 3700 V, high CAD setting, and DMS resolution enhancement to the open and individual strain gauge offset were set to −30 to 30 V. Positive electrospray mode (ESI^+^) was employed, along with an ion spray voltage of 4500 V and the following ion source parameters to achieve the fragmentation of [M−H]^+^ ions into specific products: curtain gas (35 psi), gas 1 (55 psi), gas 2 (65 psi), temperature (450 °C), and input potential (10 V). DMS-MS/MS parameters, declustering potential, collision energy, and cell exit potential, as listed in Table S3 for all phospholipids, including DMS parameters SV, DMO, and CoV, were set for each compound. The LC conditions were optimized.

Furthermore, chromatographic separation of the 63 analytes was conducted on an Exion UHPLC system (Sciex), which consisted of an ExionLC degasser, an ExionLC AD autosampler, an ExionLC AC column oven, and two ExionLC AD binary LC pump systems. The chromatographic separation was performed on a Kinetex C18 column (Phenomenex, Aschaffenburg, Germany) with a particle size of 2.1 µm, a pore size of 100 Å, and a length of 100 mm. SecurityGuard™ ULTRA Cartridge UHPLC C18 2.1 mm guard column (Phenomenex) was used in conjunction.

Chromatographic separation was achieved by employing a mobile phase consisting of 5 mM ammonium acetate buffer dissolved in water, adjusted to a pH of 2.3 with acetic acid (A), and acetonitrile/isopropanol/water (55/40/5, *v*/*v*/*v*) as the organic phase (B). The flow rate was maintained at 0.4 mL/min throughout the analysis. The gradient elution program commenced with a composition of 5% B for one minute, subsequently increasing to 60% B in 0.5 min, 80% B in 5 min, and finally reaching 100% B within another 3 min. This was maintained for a period of 2 min, after which the ratio was decreased to the initial composition of 5% B within one minute. This was followed by a period of 2 min of re-equilibration. The injection volume for all samples was 2 μL, the column oven temperature was set to 40 °C, and the autosampler temperature was maintained at 5 °C. Data acquisition and instrument control were performed using Analyst 1.6.3 software (Sciex); Multiquant 3.0.3 software (Sciex) was used for peak picking and calibration.

### 4.5. Quantitative Nuclear Magnetic Resonance (qHNMR) Spectroscopy of Stock Solutions

Aliquots of all phospholipids were weighed into 5 × 178 mm NMR tubes (USC tubes, Bruker, Faellanden, Switzerland) and dissolved in methanol-d_4_, benzene-d_6_, or acetone-d_6_ (see Table S2 for analyte-specific solvent selection). Individual phospholipids 1 H qHNMRs were acquired at 298 K on a Bruker AVANCE III 400.13 MHz system (Bruker, Rheinstetten, Germany) equipped with a Zgradient 5 mm multinuclear observation probe (BBFOplus, Bruker) and Topspin 3.0 (Bruker) software to determine the exact concentration of the stock solutions [43]. Using tyrosine as an external reference for calibration, absolute concentrations were determined by comparative integration of the isolated signals of the terminal methyl groups (e.g., 0.88–0.90 ppm in CDCl_3_, representing an integral of three for lysophospholipids and six for the remaining analytes).

### 4.6. Study Set Up

All experiments of the previously published colon cancer mouse model [15] were performed at the Chair of Nutrition and Immunology and the ZIEL Institute for Food & Health (Technical University Munich), following approval by the Committee on Animal Health Care and Use of the state of Upper Bavaria (Regierung von Oberbayern; AZ ROB-55.2-2532.Vet_02-20-58). This study included 24 mice with different tendencies to develop intestinal tumors. All mice were housed under specific pathogen-free (SPF) and germ-free (GF) conditions (12 h light/dark cycles and 24–26 °C). They were used as a humanized model to illustrate the causal effects of the intestinal lipidome in vivo in a host susceptible to developing colon cancer. At previously determined and specific age time-points, the intestinal tissue of the mice was excised and phenotypically characterized (T = tumor, NT = non-tumor). Samples were stored at −80 °C until analysis.

### 4.7. Statistical Analysis

Analysis and visualization of quantitative data were performed within the data analysis and visualization platform R (version 4.4.1) using the ropls package (1.40.0) and tidyverse (2.0.0) for calculation of the plots. The heatmap was created using the pheatmap package (1.0.13). Color scales for figures were based on the packages RColorBrewer (1.1–3), while statistical analysis was performed with rstatix (0.7.2) and labeling with the ggrepel (0.9.5) package [44,45,46,47,48,49,50]. Differences between study groups were evaluated using the Wilcoxon rank-sum test (significance threshold *p* < 0.05), since key assumptions of parametric tests were not met.

## Figures and Tables

**Table 1:** Summary of threshold values (LoD, LoQ; [ng/mg]) and validation parameters coefficient of variation (intraday and interday CVs; [%]) for individual phospholipid classes (PC, PE, PG, and PS) in tissue samples. Values represent class medians and data range.

	PC	PE	PG	PS
LoD	37.84	17.64	21.36	77.25
[ng/mg tissue]	(0.65–5990.93)	(0.19–241.54)	(0.02–257.04)	(9.5–860.76)
LoQ	126.13	58.79	71.2	257.5
[ng/mg tissue]	(2.17–19,969.78)	(0.62–805.14)	(0.08–856.8)	(31.67–2869.21)
Intraday CV	3.86	4.35	3.62	2.5
[median and range]	(1.68–6.50)	(2.40–6.25)	(1.81–5.42)	(1.78–4.22)
Interday CV	3.49	3.93	2.93	2.22
[median and range]	(1.49–5.78)	(2.13–6.63)	(1.61–4.86)	(1.58–3.75)

## Data Availability

The original contributions presented in this study are included in the article/Supplementary Materials. Further inquiries can be directed to the corresponding author.

## Supplementary Materials

The following supporting information can be downloaded at: https://www.mdpi.com/article/10.3390/molecules31030438/s1, Table S1: Analyte names of phospholipids included in method development, Table S2: Order information for the individual phospholipid references, solvents used for qNMR analysis, and stock solution concentrations determined by qNMR, Table S3: Optimized MRM transitions and DMS parameters in positive ESI mode for phospholipids using 2-propanol as DMS modifier, Table S4: Lower limits of detection and quantification, Table S5: Recovery results of different spiking levels from 25–100% of pig intestine content, Table S6: Table of validation results for individual phospholipids, Table S7: Table of the absolute concentrations [µg/mg], variance coefficients [%] and their standard deviation SD of the mouse cohort study separated into phenotypic groups (NT = non-tumor, T = tumor), Figure S1: Graphical representations of absolute concentrations [µg/mg] of phosphatidylcholines, Figure S2: Graphical representations of absolute concentrations [µg/mg] of phosphatidylethanolamines, Figure S3: Graphical representations of absolute concentrations [µg/mg] of phosphatidylglycerols, Figure S4: Graphical representations of absolute concentrations [µg/mg] of phosphatidylserines, Mandel F-value [adapted from ref. [17]].

## Abbreviations

The following abbreviations are used in this manuscript:

LC	Liquid Chromatography
UHPLC	Ultra-high-pressure/performance liquid chromatography
MS	Mass spectrometry
MS/MS	Tandem mass spectrometry
DMS	Differential ion mobility spectrometry
DMO	Differential ion mobility offset
ESI	Electrospray ionization
BHT	2.6-ditertbutyl-4-methylphenol
PC	Phosphatidylcholine
PE	Phosphatidylethanolamine
PG	Phosphatidylglycerol
PS	Phosphatidylserine
mmol	millimol
µg	microgram
mg	milligram
kg	kilogram
L	liter
°C	Degree Celsius
Å	ångström
CV	Coefficient of variance
SD	Standard deviation
T	Tumor
NT	Non-Tumor
