# Supplementary material for: Development of a High-Throughput UHPLC-DMS-MS/MS Method for Targeted Quantitation of Pertinent Phospholipid Classes in Colon Cancer
Source: Molecules. 2026 Jan 27;31(3):438. doi: 10.3390/molecules31030438 (PMC12899683; doi:10.3390/molecules31030438)
Supplement: Supplementary file 1 [file molecules-31-00438-s001.zip › molecules-4003420-supplementary.pdf]

Supplementary Materials

Development of a High-Throughput UHPLC-DMS-MS/MS Method for Targeted Quantitation of Pertinent Phospholipid Classes in Colon Cancer

Miriam Wimmer, Olivia I. Coleman, Adam Sorbie, Dirk Haller , Veronika Somoza, and Andreas Dunkel

Table S1. Analyte names of phospholipids included in method development.

Nr.	Phospholipide	IUPAC name
**Group I: phosphocholine (PC)**		
**1**	Lyso-PC(14:0/0:0)	1-Myristoyl-sn-glycero-3-phosphocholine
**2**	PC(14:0/14:0)	1 .2-Dimyristoyl-sn-glycero-3-phosphocholine
**3**	Lyso-PC(16:0/0:0)	1-Palmitoyl-sn-glycero-3-phosphocholine
**4**	PC(16:0/16:0)	1 .2-Dipalmitoyl-sn-glycero-3-phosphocholine
**5**	Lyso-PC(18:0/0:0)	1-Stearoyl-sn-glycero-3-phosphocholine
**6**	PC(18:0/18:0)	1 .2-Distearoyl-sn-glycero-3-phosphocholine
**7**	PC(18:1(11Z)/18:1(11Z))	1 .2-di-[(11Z)-octadecenoyl]-sn-glycero-3-phosphocholine
**8**	PC(18:1(11E)/18:1(11E))	1 .2-di-(11E-octadecenoyl)-sn-glycero-3-phosphocholine
**9**	PC(18:2(6Z.9Z)/18:2(6Z.9Z))	1 .2-di-(6Z .9Z-octadecadienoyl)-sn-glycero-3-phosphocholine
**10**	PC(16:0/18:0)	1-Palmitoyl-2-stearoyl-sn-glycero-3-phosphorylcholine
**11**	PC(16:0/18:1(9Z))	1-Hexadecanoyl-2-(9Z-octadecenoyl)-sn-glycero-3-phosphocholine
**12**	PC(16:0/20:4(5Z.8Z.11Z.14Z))	1-hexadecanoyl-2-(5Z .8Z .11Z .14Z-eicosatetraenoyl)-sn-glycero-3-phosphocholine
**13**	PC(18:0/16:0)	1-octadecanoyl-2-palmitoyl-sn-glycero-3-phosphocholine
**14**	PC(18:0/18:1(9Z))	1-octadecanoyl-2-(9Z-octadecenoyl)-sn-glycero-3-phosphocholine
**15**	PC(18:0/20:4(5Z.8Z.11Z.14Z))	1-octadecanoyl-2-(5Z .8Z .11Z .14Z-eicosatetraenoyl)-sn-glycero-3-phosphocholine
**16**	PC(18:0/22:6(4Z.7Z.10Z.13Z.16Z.19Z))	1-octadecanoyl-2-(4Z .7Z .10Z .13Z .16Z .19Z-docosahexaenoyl)-sn-glycero-3-phosphocholine
**17**	PC(18:1(9Z)/16:0)	1-(9Z-octadecenoyl)-2-hexadecanoyl-sn-glycero-3-phosphocholine
**18**	PC(18:1(9Z)/18:0)	1-(9Z-octadecenoyl)-2-octadecanoyl-sn-glycero-3-phosphocholine
**19**	PC(16:0/22:6(4Z.7Z.10Z.13Z.16Z.19Z))	1-hexadecanoyl-2-(4Z .7Z .10Z .13Z .16Z .19Z-docosahexaenoyl)-sn-glycero-3-phosphocholine
**Group II: phosphoethanolamine (PE)**		
**20**	Lyso-PE(14:0/0:0)	1-Myristoyl-2-hydroxy-sn-glycero-3-phosphoethanolamine
**21**	PE(14:0/14:0)	1 .2-Dimyristoyl-sn-glycero-3-phosphoethanolamine
**22**	Lyso-PE(16:0/0:0)	1-palmitoyl-sn-glycero-3-phosphoethanolamine
**23**	PE(16:0/16:0)	1 .2-Dipalmitoyl-sn-glycero-3-phosphoethanolamine
**24**	Lyso-PE(18:0/0:0)	1-octadecanoyl-sn-glycero-3-phosphoethanolamine
**25**	PE(18:0/18:0)	1 .2-Dioctadecanoyl-sn-glycero-3-phosphoethanolamine
**26**	PE(18:1(11Z)/18:1(11Z))	1 .2-di-[(11Z)-octadecenoyl]-sn-glycero-3-phosphoethanolamine
**27**	PE(18:1(11E)/18:1(11E))	1 .2-di-[(11E)-octadecenoyl]-sn-glycero-3-phosphoethanolamine
**28**	PE(16:0/18:1(9Z))	1-Hexadecanoyl-2-(9Z-octadecenoyl)-sn-glycero-3-phosphoethanolamine

**29**	PE(18:0/18:1(9Z))	1-octadecanoyl-2-(9Z-octadecenoyl)-sn-glycero-3-phosphoethanolamine
**30**	PE(18:0/20:4(5Z.8Z.11Z.14Z))	1-octadecanoyl-2-(5Z .8Z .11Z .14Z-eicosatetraenoyl)-sn-glycero-3-phosphoethanolamine
**Group III: phosphoglycerol (PG)**		
**31**	Lyso-PG(14:0/0:0)	1-Myristoyl-2-hydroxy-sn-glycero-3-phospho-(1'-rac-glycerol)
**32**	PG(14:0/14:0)	1 .2-Dimyristoyl-sn-glycero-3-(phospho-s-(1-glycerol))
**33**	Lyso-PG(16:0/0:0)	1-palmitoyl-2-hydroxy-sn-glycero-3-phospho-(1'-rac-glycerol)
**34**	PG(16:0/16:0)	1 .2-dihexadecanoyl-sn-glycero-3-phospho-(1'-sn-glycerol)
**35**	Lyso-PG(18:0/0:0)	1-stearoyl-2-hydroxy-sn-glycero-3-phospho-(1'-rac-glycerol)
**36**	PG(18:0/18:0)	1 .2-distearoyl-rac-glycero-3-phosphoglycerol
**37**	PG(18:1(11Z)/18:1(11Z))	1 .2-di(11Z-octadecenoyl)-rac-glycero-3-phospho-(1'-glycerol)
**38**	PG(18:1(11E)/18:1(11E))	1 .2-di(11E-octadecenoyl)-rac-glycero-3-phospho-(1'-glycerol)
**39**	PG(18:2(6Z.9Z)/18:2(6Z.9Z))	1 .2-di-(6Z .9Z-octadecadienoyl)-sn-glycero-3-phosphoethanolamine
**40**	PG(18:0/18:1(9Z))	1-octadecanoyl-2-(9Z-octadecenoyl)-sn-glycero-3-phospho-(1'-sn-glycerol)
**41**	PG(18:0/22:6(4Z.7Z.10Z.13Z.16Z.19Z))	1-octadecanoyl-2-(4Z .7Z .10Z .13Z .16Z .19Z-docosahexaenoyl)-sn-glycero-3-phospho-(1'-sn-glycerol)
**Group IV: phospho-L-serine (PS)**		
**44**	PS(14:0/14:0)	1 .2-ditetradecanoyl-sn-glycero-3-phosphoserine
**45**	PS(16:0/16:0)	1 .2-dihexadecanoyl-sn-glycero-3-phosphoserine
**42**	PS(18:1(11Z)/18:1(11Z))	1 .2-di(11Z-oleoyl)-sn-glycero-3-phospho-L-serine
**43**	Lyso-PS(18:1(11Z)/0:0)	1-(11Z-oleoyl)-2-hydroxy-sn-glycero-3-phospho-L-serine
**47**	PS(16:0/18:1(11Z))	1-palmitoyl-2-(11Z-oleoyl)-sn-glycero-3-phospho-L-serine
**46**	PS(18:0/18:1(11Z))	1-stearoyl-2-(11Z-oleoyl)-sn-glycero-3-phospho-L-serine
**48**	PS(18:0/18:2(9Z.12Z))	1-octadecanoyl-2-(9Z .12Z-octadecadienoyl)-sn-glycero-3-phosphoserine
**Group V: UltimateSPLASH™ ONE**		
**49**	17:0 Lyso-PC-d_5	1-heptadecanoyl-2-hydroxy-sn-glycero(d_5)-3-phosphocholine
**50**	17:0 Lyso-PE-d_5	1-heptadecanoyl-2-hydroxy-sn-glycero(d_5)-3-phosphoethanolamine
**51**	19:0 Lyso-PE-d_5	1-nonadecanoyl-2-hydroxy-sn-glycero(d_5)-3-phosphoethanolamine
**52**	17:0-14:1 PC-d_5	1-heptadecanoyl-2-(11Z-myristoleoyl)-sn-glycero(d_5)-3-phosphocholine
**53**	17:0-16:1 PC-d_5	1-heptadecanoyl-2-(11Z-palmitoleoyl)-sn-glycero(d_5)-3-phosphocholine
**54**	17:0-18:1 PC-d_5	1-heptadecanoyl-2-(11Z-oleoyl)-sn-glycero(d_5)-3-phosphocholine
**55**	17:0-20:3 PC-d_5	1-heptadecanoyl-2-(11Z .14Z .17Z-eicosatrienoyl)-sn-glycero(d_5)-3-phosphocholine
**56**	17:0-22:4 PC-d_5	1-heptadecanoyl-2-(5Z .8Z .11Z .14Zdocosatetraenoyl)-sn-glycero(d_5)-3-phosphocholine
**57**	17:0-14:1 PE-d_5	1-heptadecanoyl-2-(11Z-myristoleoyl)-sn-glycero(d_5)-3-phosphoethanolamine
**58**	17:0-16:1 PE-d_5	1-heptadecanoyl-2-(11Z-palmitoleoyl(11Z)-sn-glycero(d_5)-3-phosphoethanolamine
**59**	17:0-18:1 PE-d_5	1-heptadecanoyl-2-(11Z-oleoyl)-sn-glycero(d_5)-3-phosphoethanolamine

**60**	17:0-20:3 PE-d_5	1-heptadecanoyl-2-(11Z .14Z .17Z-eicosatrienoy)l-sn-glycero(d_5)-3-phosphoethanolamine
**61**	17:0-22:4 PE-d_5	1-heptadecanoyl-2-(5Z .8Z .11Z .14Z-docosatetraenoyl)-sn-glycero(d_5)-3-phosphoethanolamine
**62**	17:0-16:1 PG-d_5	1-heptadecanoyl-2-(11Z-palmitoleoyl)-sn-glycero(d_5)-3-phospho-(1'-rac-glycerol)
**63**	17:0-18:1 PS-d_5	1-heptadecanoyl-2-l(11Z-oleoy)-sn-glycero(d_5)-3-phospho- L-serine
**Group VI: isotopic internal work-up solution**		
**64**	C16:0-16.16.16-d_3	Hexadecanoic acid-16.16.16-d_3
**65**	C18:0-9.9.10.10-d_4	Octadecanoic acid-9.9.10.10-d_4

Table S2. Order information for the individual phospholipid references at Avanti Research, solvents used for qNMR analysis, and stock solution concentrations determined by qNMR.

	Order number	Shipping format	Purity manufacturer	MW [mg/mmol]	qNMR solvent	qNMR [mmol/L]
**a) Phosphatidylcholine**						
**LPC(14:0/0:0)**	855575C-200mg	Chloroform	>99% LPC; may contain up to 10% of the 2-LPC isomer	467.30	MeOH-d_4	**2.33**
**LPC(16:0/0:0)**	855675C-25mg	powder	>99% LPC; may contain up to 10% of the 2-LPC isomer	495.33	MeOH-d_4	**2.38**
**LPC(18:0/0:0)**	855775C-25mg	Chloroform	>99% LPC; may contain up to 10% of the 2-LPC isomer	523.36	MeOH-d_4	**1.42**
**PC(14:0/14:0)**	850345C-25mg	Chloroform	>99%	677.50	MeOH-d_4	**1.33**
**PC(16:0/16:0)**	850355C-25mg	Chloroform	>99%	733.56	MeOH-d_4	**1.48**
**PC(18:0/18:0)**	850365C-25mg	Chloroform	>99%	789.63	MeOH-d_4	**1.55**
**PC(18:1(Z)/18:1(Z))**	850375C-25mg	Chloroform	>99%	785.59	MeOH-d_4	**1.30**
**PC(18:1(E)/18:1(E))**	850376C-25mg	Chloroform	>99%	785.59	MeOH-d_4	**1.20**
**PC(18:2/18:2)**	850385C-25mg	Chloroform	>99%	781.56	MeOH-d_4	**1.47**
**PC(16:0/18:1)**	850457C-25mg	Chloroform	>99%	760.08	MeOH-d_4	**0.59**
**PC(16:0/18:0)**	850456C-25mg	Chloroform	>99%	762.09	MeOH-d_4	**0.67**
**PC(16:0/20:4)**	850459C-25mg	Chloroform	>99%	782.08	MeOH-d_4	**0.12**
**PC(16:0/22:6)**	850461C-25mg	Chloroform	>99%	806.10	MeOH-d_4	**0.13**
**PC(18:0/16:0)**	850465P-25mg	powder	>99%	762.09	MeOH-d_4	**0.78**
**PC(18:0/18:1)**	850467C-25mg	Chloroform	>99%	788.13	MeOH-d_4	**0.67**
**PC(18:0/20:4)**	850469C-25mg	Chloroform	>99%	810.13	MeOH-d_4	**0.13**
**PC(18:0/22:6)**	850472C-25mg	Chloroform	>99%	834.16	MeOH-d_4	**0.64**
**PC(18:1/16:0)**	850475C-25mg	Chloroform	>99%	760.08	MeOH-d_4	**0.58**
**PC(18:1/18:0)**	850476C-25mg	Chloroform	>99%	788.13	MeOH-d_4	**0.67**
**b) Phosphoethanolamine**						
**LPE(14:0/0:0)**	856735P-25mg	powder	>99% LPE; may contain up to 10% of the 2-LPE isomer	425.25	MeOH-d_4	**2.14**
**LPE(16:0/0:0)**	856705P-25mg	powder	>99% LPE; may contain up to 10% of the 2-LPE isomer	453.29	MeOH-d_4	**1.02**
**LPE(18:0/0:0)**	856715P-25mg	powder	>99% LPE; may contain up to 10% of the 2-LPE isomer	481.32	DMSO-d_6	**2.15**
**PE(14:0/14:0)**	850745P-25mg	powder	>99%	635.45	DMSO-d_6	**0.10**

**PE(16:0/16:0)**	850705P-25mg	powder	>99%	691.52	Cholorform-d_1	**0.85**
**PE(18:0/18:0)**	850715P-25mg	powder	>99%	747.58	DMSO-d_6	**0.04**
**PE(18:1(Z)/18:1(Z))**	850725C-25mg	Chloroform	>99%	743.55	MeOH-d_4	**1.61**
**PE(18:1(E)/18:1(E))**	850726C-25mg	Chloroform	>99%	743.55	MeOH-d_4	**0.72**
**PE(16:0/18:1)**	850757C-25mg	Chloroform	>99%	718.00	MeOH-d_4	**0.71**
**PE(18:0/18:1)**	850758P-25mg	powder	>99%	746.05	MeOH-d_4	**0.67**
**PE(18:0/20:4)**	850804C-25mg	Chloroform	>99%	768.06	MeOH-d_4	**0.13**
**c) Phosphatidylglycerol**						
**LPG(14:0/0:0)**	858120P-500mg	powder	>99% LPG; may contain up to 10% of the 2-LPG isomer	478.23	MeOH-d_4	**2.15**
**LPG(16:0/0:0)**	858122P-500mg	powder	>99% LPG; may contain up to 10% of the 2-LPG isomer	506.26	MeOH-d_4	**1.76**
**LPG(18:0/0:0)**	858124P-500mg	powder	>99% LPG; may contain up to 10% of the 2-LPG isomer	534.29	MeOH-d_4	**2.00**
**PG(14:0/14:0)**	840445P-25mg	powder	>99%	688.43	MeOH-d_4	**1.79**
**PG(16:0/16:0)**	840455P-25mg	powder	>99%	744.49	MeOH-d_4	**1.30**
**PG(18:0/18:0)**	840465P-25mg	powder	>99%	800.55	DMSO-d_6	**0.33**
**PG(18:1(Z)/18:1(Z))**	840475C-25mg	Chloroform	>99%	796.52	MeOH-d_4	**1.55**
**PG(18:1(E)/18:1(E))**	840477P-25mg	powder	>99%	796.52	MeOH-d_4	**1.35**
**PG(18:2/18:2)**	840485C-25mg	Chloroform	>99%	792.49	MeOH-d_4	**1.12**
**PG(18:0/18:1)**	840503C-25mg	Chloroform	>99%	792.08	MeOH-d_4	**0.63**
**PG(18:0/22:6)**	840506C-25mg	Chloroform	>99%	799.04	MeOH-d_4	**0.14**
**d) Phosphatidylserine**						
**LPS(18:1/0:0)**	858143C-5mg	Chloroform	>99% LPS; may contain up to 10% of the 2-LPS isomer	545.27	MeOH-d_4	**2.10**
**PS(14:0/14:0)**	840033P-10mg	powder	>99%	701.42	MeOH-d_4	**1.39**
**PS(16:0/16:0)**	840037P-10mg	powder	>99%	757.49	MeOH-d_4	**0.76**
**PS(18:1(Z)/18:1(Z))**	840035C-10mg	Chloroform	>99%	809.52	MeOH-d_4	**1.18**
**PS(16:0/18:1)**	840034C-10mg	Chloroform	>99%	797.03	MeOH-d_4	**0.67**
**PS(18:0/18:1)**	840039C-10mg	Chloroform	>99%	845.07	MeOH-d_4	**0.62**
**PS(18:0/18:2)**	840063C-10mg	Chloroform	>99%	783.99	MeOH-d_4	**0.63**

Table S3. Optimized MRM transitions and MS parameters (declustering potential = DP, collision energy = CE, cell exit potential = CXP) and DMS parameters (SV = separation voltage, CoV = compensation voltage, DMO = differential mobility offset) in positive ESI mode for phospholipids using 2-propanol as DMS modifier.

Component name	Internal standard for quantitation	Q1 (m/z)	Q3 (m/z)	Time (min)	DP (V)	CE (V)	CXP (V)	SV (V)	CoV (V)	DMO (V)
**Group I: Phosphatidylcholines (PC)**										
1[Q]	49	468.22	184.10	3.01	121	10	33	22	5	-3
1[Id]	49	468.22	104.10	3.01	121	10	27	12	5	-3
2[Q]	49	678.39	184.10	5.47	1	10	37	16	5	-3
2[Id]	49	678.39	124.90	5.47	1	10	93	14	5	-3
3[Q]	49	496.26	184.00	3.29	96	10	33	22	6	-3
3[Id]	49	496.26	104.10	3.29	96	10	29	12	6	-3
4[Q]	49	734.47	184.00	7.38	46	10	37	22	6	-3
4[Id]	49	734.47	125.00	7.38	46	10	99	14	6	-3
5[Q]	49	524.32	184.10	3.67	106	10	33	22	6	-3
5[Id]	49	524.32	104.00	3.67	106	10	29	12	6	-3
6[Q]	49	790.54	184.00	8.01	96	10	39	20	6	-3
6[Id]	49	790.54	125.00	8.01	96	10	117	14	6	-3
7[Q]	52	786.52	184.10	7.42	91	10	41	22	8.2	-50
7[Id]	52	786.50	311.44	7.42	91	10	39	24	8.2	-50
8[Q]	52	786.51	124.90	7.84	96	10	129	12	5.8	-42
8[Id]	52	786.51	662.80	7.84	96	10	13	12	5.8	-42
9[Q]	54	782.49	184.10	6.51	86	10	39	22	7	-3
9[Id]	54	782.49	124.90	6.51	86	10	113	14	7	-3
10[Q]	54	762.46	184.10	8.1	151	10	37	22	6.6	-26
10[Id]	54	762.46	496.20	8.1	151	10	37	14	6.6	-26
11[Q]	54	761.15	184.10	7.4	1	10	39	22	5.6	-46
11[Id]	54	761.15	185.10	7.4	1	10	37	18	5.6	-46
12[Q]	54	782.43	184.10	6.51	136	10	39	22	7	-3
12[Id]	54	782.43	125.00	6.51	136	10	109	14	7	-3
13[Q]	54	806.43	184.00	6.62	96	10	39	22	6	-3
13[Id]	54	806.43	125.00	6.62	96	10	113	14	6	-3
14[Q]	54	762.46	184.10	7.6	1	10	39	22	5.8	-50
14[Id]	54	762.46	524.30	7.6	1	10	37	16	5.8	-50
15[Q]	54	788.48	184.10	8.43	1	10	39	22	5.6	-49
15[Id]	54	788.48	524.30	8.43	1	10	39	16	5.6	-49
16[Q]	55	810.48	184.00	7.41	131	10	39	22	7	-3
16[Id]	55	810.48	627.50	7.41	131	10	45	18	7	-3
17[Q]	56	834.48	184.10	6.66	86	10	39	22	6	-3
17[Id]	56	834.48	125.00	6.66	86	10	115	14	6	-3
18[Q]	54	761.44	184.10	7	1	10	37	10	6.2	-50
18[Id]	54	761.44	125.00	7	1	10	107	14	6.2	-50
19[Q]	54	789.47	126.00	7.85	121	10	113	14	9.2	-41
19[Id]	54	789.47	185.10	7.85	121	10	39	22	9.2	-41
**Group II: Phosphatidylethanolamine (PE)**										

20^{Q}	50	426.16	285.20	2.95	56	10	23	16	-9	-3
20^{Id}	50	426.16	408.20	2.95	56	10	15	20	-9	-3
21^{Q}	50	636.38	495.40	5.14	86	10	27	14	-1	-3
21^{Id}	50	636.38	211.20	5.14	86	10	39	12	-1	-3
22^{Q}	50	454.19	313.20	3.22	51	10	25	16	-7	-3
22^{Id}	50	454.19	436.30	3.22	51	10	17	6	-7	-3
23^{Q}	50	692.43	551.50	6.53	76	10	27	16	0	-3
23^{Id}	50	692.43	239.30	6.53	76	10	41	16	0	-3
24^{Q}	50	482.31	341.20	3.55	71	10	25	18	-6	-3
24^{Id}	50	482.31	464.30	3.55	71	10	17	24	-6	-3
25^{Q}	51	748.58	607.60	7.5	56	10	31	8	1	-3
25^{Id}	51	748.58	686.70	7.5	56	10	7	20	1	-3
26^{Q}	59	744.51	603.50	6.73	26	10	31	18	-0.8	-19
26^{Id}	59	744.51	620.40	6.73	26	10	13	32	-0.8	-19
27^{Q}	59	744.46	265.20	6.9	156	10	45	14	4.8	-25
27^{Id}	59	744.46	308.39	6.9	156	10	53	30	4.8	-25
28^{Q}	59	718.41	577.50	6.61	1	10	29	10	1	-3
28^{Id}	59	718.41	265.20	6.61	1	10	43	14	1	-3
29^{Q}	59	746.43	605.50	7.25	1	10	31	8	2	-3
29^{Id}	59	746.43	265.20	7.25	1	10	45	14	2	-3
30^{Q}	60	768.43	627.50	6.74	86	10	29	18	2	-3
30^{Id}	60	768.43	341.20	6.74	86	10	43	18	2	-3
**Group III: Phospahtidylglycreol (PG)**										
31^{Q}	49	479.16	195.00	2.74	111	10	31	10	-4	-3
31^{Id}	49	479.16	285.20	2.74	111	10	35	14	-4	-3
32^{Q}	62	689.32	195.00	4.72	91	10	35	24	1	-3
32^{Id}	62	689.32	495.40	4.72	91	10	39	24	1	-3
33^{Q}	49	507.20	195.00	2.94	126	10	33	24	-2	-3
33^{Id}	49	507.20	313.30	2.94	126	10	35	16	-2	-3
34^{Q}	62	745.39	195.00	5.62	126	10	39	22	3	-3
34^{Id}	62	745.39	551.40	5.62	126	10	41	16	3	-3
35^{Q}	49	535.22	194.90	3.17	131	10	33	10	-1	-3
35^{Id}	49	535.22	341.20	3.17	131	10	37	18	-1	-3
36^{Q}	62	801.35	195.00	6.55	1	10	39	22	3	-3
36^{Id}	62	801.35	723.20	6.55	1	10	13	20	3	-3
37^{Q}	62	797.43	195.00	5.73	176	10	41	22	2.6	-26
37^{Id}	62	797.43	643.59	5.73	176	10	43	18	2.6	-26
38^{Q}	62	797.44	194.90	5.87	121	10	43	18	3.4	-25
38^{Id}	62	797.44	779.60	5.87	121	10	11	14	3.4	-25
39^{Q}	62	793.40	195.00	4.83	86	10	39	10	2	-3
39^{Id}	62	793.40	621.40	4.83	86	10	39	18	2	-3
40^{Q}	62	799.40	194.90	6.13	161	10	39	10	3	-3
40^{Id}	62	799.40	605.50	6.13	161	10	43	8	3	-3
41^{Q}	62	845.41	673.50	5.67	96	10	43	20	3	-3
41^{Id}	62	845.41	195.00	5.67	96	10	45	10	3	-3
**Group IV: Phosphatidylserine (PS)**										
42^{Q}	63	702.32	208.00	4.78	101	10	35	24	1	-3

42[Id]	63	702.32	495.40	4.78	101	10	37	14	1	-3
43[Q]	63	758.36	208.00	5.78	101	10	37	24	2	-3
43[Id]	63	758.36	551.50	5.78	101	10	41	16	2	-3
44[Q]	63	810.43	208.00	5.95	161	10	41	12	2	-3
44[Id]	63	810.43	603.50	5.95	161	10	43	18	2	-3
45[Q]	63	546.19	459.10	3.01	146	10	33	22	-3	-3
45[Id]	63	546.19	339.30	3.01	146	10	39	18	-3	-3
46[Q]	63	784.37	208.00	5.84	116	10	37	24	2	-3
46[Id]	63	784.37	577.40	5.84	116	10	43	16	2	-3
47[Q]	63	812.40	208.00	5.88	136	10	39	12	2	-3
47[Id]	63	812.40	605.50	5.88	136	10	43	18	2	-3
48[Q]	63	810.39	208.00	5.96	141	10	39	24	3	-3
48[Id]	63	810.39	603.40	5.96	141	10	43	18	3	-3
**Group V: isotopic labeled standard**										
49[Q]		515.29	184.10	3.44	96	10	35	22	6	-3
49[Id]		515.29	104.00	3.44	96	10	29	12	6	-3
50[Q]		473.30	332.20	3.33	71	10	25	18	-6	-3
50[Id]		473.30	200.00	3.33	71	10	43	10	-6	-3
51[Q]		501.31	360.20	3.72	61	10	25	18	-4	-3
51[Id]		501.31	484.30	3.72	61	10	13	14	-4	-3
52[Q]		723.42	184.10	5.87	76	10	37	22	6	-3
52[Id]		723.42	125.00	5.87	76	10	107	12	6	-3
53[Q]		751.47	184.10	6.39	106	10	37	22	7	-3
53[Id]		751.47	124.90	6.39	106	10	113	14	7	-3
54[Q]		779.49	184.10	7	76	10	37	22	7	-3
54[Id]		779.49	124.90	7	76	10	111	14	7	-3
55[Q]		803.50	184.00	6.74	116	10	39	22	7	-3
55[Id]		803.50	125.00	6.74	116	10	117	14	7	-3
56[Q]		829.50	184.10	6.93	111	10	41	18	8	-3
56[Id]		829.50	569.40	6.93	111	10	27	16	8	-3
57[Q]		681.41	540.50	5.51	66	10	29	16	0	-3
57[Id]		681.41	664.40	5.51	66	10	19	20	0	-3
58[Q]		709.38	568.50	5.91	86	10	29	16	1	-3
58[Id]		709.38	192.10	5.91	86	10	33	24	1	-3
59[Q]		737.42	596.40	6.34	91	10	33	18	1	-3
59[Id]		737.42	308.30	6.34	91	10	49	16	1	-3
60[Q]		761.43	620.40	6.18	106	10	31	18	3	-3
60[Id]		761.43	184.10	6.18	106	10	37	22	3	-3
61[Q]		787.50	646.40	6.34	66	10	35	18	3	-3
61[Id]		787.50	598.60	6.34	66	10	23	8	3	-3
62[Q]		762.43	621.60	6.18	76	10	33	8	2	-3
62[Id]		762.43	184.20	6.18	76	10	39	8	2	-3
63[Q]		803.47	184.10	6.73	61	10	39	22	6	-3
63[Id]		803.47	125.10	6.73	61	10	117	12	6	-3
64[Q]		259.03	259.10	3.45	111	10	7	30	-7	-3
64[Id]		259.03	184.90	3.45	111	10	21	18	-7	-3
65[Q]		288.22	288.20	2.94	36	10	7	16	4	-3
65[Id]		288.22	270.20	2.94	36	10	11	14	4	-3

Q represents quantifier, Id represents identifier

Table S4. Lower limits of detection and quantification (LoD/LoQ) in ng/mg with regression (R^2) and the recovery rate S in %.

		[ng/mg]	R^2	S [%]
**LPC(14:0/0:0)**	LoD	16.47	0.99	100.7
	LoQ	54.92	± 0.006	
**LPC(16:0/0:0)**	LoD	1.39	0.99	109.69
	LoQ	4.63	± 0.003	
**LPC(18:0/0:0)**	LoD	0.65	0.99	92.43
	LoQ	2.17	± 0.002	
**PC(14:0/14:0)**	LoD	4.69	0.99	105.2
	LoQ	15.62	± 0.002	
**PC(16:0/16:0)**	LoD	137.63	0.99	100.05
	LoQ	458.77	± 0.001	
**PC(18:0/18:0)**	LoD	15.49	0.99	101.75
	LoQ	51.63	± 0.003	
**PC(18:1(Z)/18:1(Z))**	LoD	5221.52	0.97	114.81
	LoQ	17405.06	± 0.034	
**PC(18:1(E)/18:1(E))**	LoD	7.31	0.99	91.93
	LoQ	24.36	± 0.002	
**PC(18:2/18:2)**	LoD	164.33	0.99	92.04
	LoQ	547.76	± 0.004	
**PC(16:0/18:0)**	LoD	165.26	0.99	107.6
	LoQ	550.88	± 0.0045	
**PC(16:0/18:1)/PC(18:1/16:0)**	LoD	3658.88	0.99	104.7
	LoQ	12196.25	± 0.003	
**PC(16:0/20:4)**	LoD	21.21	0.99	108.51
	LoQ	70.69	± 0.001	
**PC(16:0/22:6)**	LoD	48.77	0.99	100.37
	LoQ	162.55	± 0.0003	
**PC(18:0/16:0)**	LoD	342.32	0.99	100.17
	LoQ	1141.06	± 0.004	
**PC(18:0/18:1)**	LoD	26.91	0.99	95.29
	LoQ	89.70	± 0.001	
**PC(18:0/20:4)**	LoD	5.17	0.99	107.02
	LoQ	17.25	± 0.001	
**PC(18:0/22:6)**	LoD	5990.93	0.99	96.15
	LoQ	19969.78	± 0.001	
**PC(18:1/18:0)**	LoD	10143.70	0.99	94.22
	LoQ	33812.32	± 0.011	
**LPE(14:0/0:0)**	LoD	1.04	0.99	110.61
	LoQ	3.46	± 0.002	
**LPE(16:0/0:0)**	LoD	1.27	0.99	89.72
	LoQ	4.24	± 0.003	
**LPE(18:0/0:0)**	LoD	33.77	0.99	101.59

	LoQ	112.56	± 0.006	
**PE(14:0/14:0)**	LoD	0.19	0.99	108.48
	LoQ	0.62	± 0.001	
**PE(16:0/16:0)**	LoD	16.06	0.98	106.14
	LoQ	53.55	± 0.007	
**PE(18:0/18:0)**	LoD	241.54	0.98	96.24
	LoQ	805.14	± 0.009	
**PE(18:1(Z)/18:1(Z))**	LoD	65.73	0.98	94.17
	LoQ	219.11	± 0.015	
**PE(18:1(E)/18:1(E))**	LoD	211.48	0.99	99.17
	LoQ	704.95	± 0.0002	
**PE(16:0/18:1)**	LoD	17.64	0.99	92.39
	LoQ	58.79	± 0.001	
**PE(18:0/18:1)**	LoD	12.26	0.99	90.68
	LoQ	40.86	± 0.002	
**PE(18:0/20:4)**	LoD	19.36	0.99	105.45
	LoQ	64.52	± 0.007	
**LPG(14:0/0:0)**	LoD	0.02	0.99	86.12
	LoQ	0.08	-	
**LPG(16:0/0:0)**	LoD	11.88	0.99	114.73
	LoQ	39.60	± 0.001	
**LPG(18:0/0:0)**	LoD	4.05	0.99	101.44
	LoQ	13.49	± 0.001	
**PG(14:0/14:0)**	LoD	3.66	0.99	70.51
	LoQ	12.21	± 0.001	
**PG(16:0/16:0)**	LoD	21.36	0.99	105.19
	LoQ	71.20	± 0.001	
**PG(18:0/18:0)**	LoD	22.48	0.99	108.75
	LoQ	74.94	± 0.001	
**PG(18:1(Z)/18:1(Z))**	LoD	37.18	0.99	92.95
	LoQ	123.94	± 0.002	
**PG(18:1(E)/18:1(E))**	LoD	27.18	0.99	86.67
	LoQ	90.60	-	
**PG(18:2/18:2)**	LoD	13.06	0.98	114.87
	LoQ	43.54	± 0.013	
**PG(18:0/18:1)**	LoD	89.09	0.98	88.62
	LoQ	296.97	± 0.015	
**PG(18:0/22:6)**	LoD	257.04	0.99	75.04
	LoQ	856.80	± 0.001	
**LPS(18:1/0:0)**	LoD	16.66	0.99	90.52
	LoQ	55.53	± 0.009	
**PS(14:0/14:0)**	LoD	9.50	0.97	104.71
	LoQ	31.67	± 0.015	
**PS(16:0/16:0)**	LoD	104.76	0.99	97.82
	LoQ	349.19	± 0.008	
**PS(18:1(Z)/18:1(Z))**	LoD	276.05	0.99	89.44

	LoQ	920.15	± 0.003	
**PS(16:0/18:1)**	LoD	45.69	0.97	89.5
	LoQ	152.29	± 0.005	
**PS(18:0/18:1)**	LoD	860.76	0.97	89.5
	LoQ	2869.21	± 0.005	
**PS(18:0/18:2)**	LoD	77.25	0.99	101.29
	LoQ	257.50	± 0.001	

Table S5. Recovery results of different spiking levels from 25% - 100% of pig intestine content.

		**Recovery level**			
		**25%**	**50%**	**75%**	**100%**
**LPC(14:0/0:0)**	**Recovery [%]**	94.24	97.08	101.11	99.11
	**RSD**	±4.21	±5.71	±9.03	±10.27
**LPC(16:0/0:0)**	**Recovery [%]**	106.12	109.71	100.52	102.69
	**RSD**	±4.54	±1.76	±10.84	±10.19
**LPC(18:0/0:0)**	**Recovery [%]**	106.69	104.29	104.81	105.18
	**RSD**	±3.50	±8.308	±7.42	±7.36
**PC(14:0/14:0)**	**Recovery [%]**	100.94	100.44	96.83	94.32
	**RSD**	±7.55	±5.173	±14.50	±11.25
**PC(16:0/16:0)**	**Recovery [%]**	98.39	107.69	92.11	96.54
	**RSD**	±11.01	±3.83	±6.01	±8.64
**PC(18:0/18:0)**	**Recovery [%]**	102.54	102.34	101.78	94.38
	**RSD**	±7.24	±7.77	±10.43	±6.66
**PC(18:1(Z)/18:1(Z))**	**Recovery [%]**	95.4	101.56	103.78	102.83
	**RSD**	±6.73	±6.56	±10.78	±6.11
**PC(18:1(E)/18:1(E))**	**Recovery [%]**	97.62	98.13	94.85	93.74
	**RSD**	±2.81	±8.94	±8.46	±7.78
**PC(18:2/18:2)**	**Recovery [%]**	96.23	104.38	101.09	98.28
	**RSD**	±4.21	±4.94	±9.27	±11.33
**PC(16:0/18:0)**	**Recovery [%]**	98.16	103.88	100.54	101.07
	**RSD**	±8.38	±4.08	±8.33	±7.56
**PC(16:0/18:1)/PC(18:1/16:0)**	**Recovery [%]**	107.73	102.07	103.38	103.95
	**RSD**	±5.21	±7.24	±2.87	±5.97
**PC(16:0/20:4)**	**Recovery [%]**	91.53	104.56	104.39	102.61
	**RSD**	±4.38	±4.23	±7.65	±7.86
**PC(16:0/22:6)**	**Recovery [%]**	89.4	93.57	98.24	94.06
	**RSD**	±3.02	±5.09	±12.13	±7.49
**PC(18:0/16:0)**	**Recovery [%]**	93.4	107.49	102.1	101.58
	**RSD**	±4.82	±6.35	±10.68	±9.15
**PC(18:0/18:1)**	**Recovery [%]**	98.99	101.46	96.45	91.34
	**RSD**	±5.67	±10.16	±11.13	±7.49
**PC(18:0/20:4)**	**Recovery [%]**	89.82	100.59	93.24	99.33
	**RSD**	±3.86	±6.46	±6.72	±7.52
**PC(18:0/22:6)**	**Recovery [%]**	98.72	108.23	99.89	103.32
	**RSD**	±7.17	±5.24	±3.95	±6.72
**PC(18:1/18:0)**	**Recovery [%]**	103.97	100.59	102.77	101.64
	**RSD**	±9.49	±5.33	±6.68	±6.23
**LPE(14:0/0:0)**	**Recovery [%]**	106.59	101.05	102.72	105.2
	**RSD**	±6.91	±8.81	±9.43	±8.44
**LPE(16:0/0:0)**	**Recovery [%]**	91.68	98.53	98.85	98.32
	**RSD**	±8.05	±6.30	±10.21	±13.40
**LPE(18:0/0:0)**	**Recovery [%]**	95.29	102.51	99.48	96.88
	**RSD**	±5.04	±6.73	±10.06	±9.62
**PE(14:0/14:0)**	**Recovery [%]**	96.22	102.55	111.44	103.34

	**RSD**	±8.07	±7.96	±2.80	±5.31
**PE(16:0/16:0)**	**Recovery [%]**	102.71	107.83	86.67	98.58
	**RSD**	±9.56	±7.63	±12.34	±6.90
**PE(18:0/18:0)**	**Recovery [%]**	104.21	102.64	94.87	97.24
	**RSD**	±8.52	±9.74	±17.01	±5.84
**PE(18:1(Z)/18:1(Z))**	**Recovery [%]**	97.07	104.18	96.87	94.03
	**RSD**	±7.72	±7.61	±5.21	±7.6
**PE(18:1(E)/18:1(E))**	**Recovery [%]**	108.3	103.5	97.67	99.97
	**RSD**	±6.07	±6.04	±8.89	±7.34
**PE(16:0/18:1)**	**Recovery [%]**	100.32	103.07	89.91	101.3
	**RSD**	±9.96	±8.77	±10.75	±10.41
**PE(18:0/18:1)**	**Recovery [%]**	95.5	103.99	101.97	107.57
	**RSD**	±8.77	±6.31	±7.97	±6.41
**PE(18:0/20:4)**	**Recovery [%]**	108.62	104.41	103.59	103.05
	**RSD**	±18.20	±7.33	±11.74	±10.36
**LPG(14:0/0:0)**	**Recovery [%]**	96.84	106.43	106.21	103.57
	**RSD**	±4.82	±8.41	±8.61	±6.45
**LPG(16:0/0:0)**	**Recovery [%]**	98.47	104.38	108.7	103.92
	**RSD**	±5.41	±6.73	±4.08	±5.05
**LPG(18:0/0:0)**	**Recovery [%]**	102.77	103.09	102.21	97.2
	**RSD**	±4.85	±8.91	±9.64	±7.25
**PG(14:0/14:0)**	**Recovery [%]**	92.63	103.39	101.19	104.19
	**RSD**	±6.54	±8.58	±9.42	±4.78
**PG(16:0/16:0)**	**Recovery [%]**	101.6	98.14	100.23	101.32
	**RSD**	±7.19	±8.24	±11.76	±7.35
**PG(18:0/18:0)**	**Recovery [%]**	102.66	99.71	98.59	100.92
	**RSD**	±7.46	±11.45	±9.33	±8.74
**PG(18:1(Z)/18:1(Z))**	**Recovery [%]**	98.16	99.76	91.95	96.3
	**RSD**	±8.41	±6.21	±8.42	±8.96
**PG(18:1(E)/18:1(E))**	**Recovery [%]**	98.37	99.69	97.11	96.31
	**RSD**	±5.84	±7.07	±4.20	±8.16
**PG(18:2/18:2)**	**Recovery [%]**	100.97	99.85	94.63	105.46
	**RSD**	±9.13	±6.52	±9.35	±7.23
**PG(18:0/18:1)**	**Recovery [%]**	97.15	97.2	102.09	100.7
	**RSD**	±12.66	±10.01	±6.31	±9.05
**PG(18:0/22:6)**	**Recovery [%]**	101.63	100.38	104	106.23
	**RSD**	±11.32	±9.33	±8.32	±5.47
**LPS(18:1/0:0)**	**Recovery [%]**	99.89	105.65	96.34	101.14
	**RSD**	±9.38	±3.62	±10.17	±6.10
**PS(14:0/14:0)**	**Recovery [%]**	100.4	99.08	103.81	103.3
	**RSD**	±9.70	±6.25	±8.77	±7.81
**PS(16:0/16:0)**	**Recovery [%]**	81.77	95.74	101.97	92.88
	**RSD**	±5.83	±7.30	±14.18	±20.25
**PS(18:1(Z)/18:1(Z))**	**Recovery [%]**	100.39	105.65	99.15	104.69
	**RSD**	±5.54	±8.34	±8.69	±8.83
**PS(16:0/18:1)**	**Recovery [%]**	94.1	100	84.38	102.44

	**RSD**	±15.12	±5.99	±17.84	±7.49
**PS(18:0/18:1)**	**Recovery [%]**	106.19	102.81	98.67	102.9
	**RSD**	±8.20	±8.89	±9.09	±9.25
**PS(18:0/18:2)**	**Recovery [%]**	93.83	102.75	97.78	103.96
	**RSD**	±12.78	±7.04	±15.71	±6.22

Table S6. Table of validation results for individual (a) Phosphatidylcholines (PC), (b) Phosphatidylethanolamines (PE), (c) Phosphatidylglycerols (PG), (d) Phosphatidylserines (PS), separated by processing and measurement periods of the individual phospholipids using the percentage coefficient of variation CV [%] with standard deviation SD_{CV} and the absolute concentration with standard deviation SD_V. Concentration of the standard stock solution c added for processing is given in [µmol/l].

		c [µmol/l]		intraday	interday
(a)	LPC(14:0/0:0)	4.24	value [µg/mg]	3.29	3.63
			SD_V	±0.40	±0.37
			CV [%]	5.63	5.02
			SD_{CV}	±2.36	±1.22
	LPC(16:0/0:0)	126.26	value [µg/mg]	51.65	61.91
			SD_V	±3.94	±3.70
			CV [%]	3.88	3.50
			SD_{CV}	±1.44	±0.30
	LPC(18:0/0:0)	94.27	value [µg/mg]	29.68	35.61
			SD_V	±2.38	±2.46
			CV [%]	4.56	4.12
			SD_{CV}	±1.58	±1.79
	PC(14:0/14:0)	2.08	value [µg/mg]	1.33	1.59
			SD_V	±0.12	±0.08
			CV [%]	3.83	3.48
			SD_{CV}	±1.94	±1.60
	PC(16:0/16:0)	160.83	value [µg/mg]	140.39	167.87
			SD_V	±12.23	±8.55
			CV [%]	2.92	2.48
			SD_{CV}	±2.00	±0.31
	PC(18:0/18:0)	3.84	value [µg/mg]	78.79	94.38
			SD_V	±6.35	±5.30
			CV [%]	2.92	2.59
			SD_{CV}	±1.10	±0.58
	PC(18:1(Z)/18:1(Z))	50.44	value [µg/mg]	78.80	94.15
			SD_V	±6.51	±3.95
			CV [%]	4.69	4.16
			SD_{CV}	±1.73	±0.65
	PC(18:1(E)/18:1(E))	138.92	value [µg/mg]	109.40	130.81
			SD_V	±8.52	±6.19
			CV [%]	3.80	3.37
			SD_{CV}	±1.66	±0.27
	PC(18:2/18:2)	95.10	value [µg/mg]	55.18	65.87
			SD_V	±5.10	±3.35
			CV [%]	3.49	3.10
			SD_{CV}	±1.96	±1.41
	PC(16:0/18:0)	22.09	value [µg/mg]	57.27	68.69
			SD_V	±4.29	±4.07

			**CV [%]**	2.24	1.99
			**SDcv**	±1.21	±0.46
	**PC(16:0/18:1)/ PC(18:1/16:0)**	**403.76**	**value [µg/mg]**	251.33	296.23
			**SDv**	±31.85	±15.20
			**CV [%]**	4.78	4.58
			**SDcv**	±1.73	±0.83
	**PC(16:0/20:4)**	**1.96**	**value [µg/mg]**	45.28	54.44
			**SDv**	±2.64	±3.45
			**CV [%]**	4.21	3.74
			**SDcv**	±0.97	±1.20
	**PC(16:0/22:6)**	**120.00**	**value [µg/mg]**	10.78	12.98
			**SDv**	±0.83	±0.94
			**CV [%]**	1.68	1.49
			**SDcv**	±1.02	±0.25
	**PC(18:0/16:0)**	**179.36**	**value [µg/mg]**	191.06	229.32
			**SDv**	±12.67	±13.18
			**CV [%]**	5.41	4.81
			**SDcv**	±2.46	±2.15
	**PC(18:0/18:1)**	**74.63**	**value [µg/mg]**	71.71	86.22
			**SDv**	±5.32	±5.68
			**CV [%]**	1.78	1.58
			**SDcv**	±1.21	±0.68
	**PC(18:0/20:4)**	**72.49**	**value [µg/mg]**	0.55	0.65
			**SDv**	±0.04	±0.03
			**CV [%]**	2.05	1.83
			**SDcv**	±0.72	±0.03
	**PC(18:0/22:6)**	**87.80**	**value [µg/mg]**	2384.78	2866.78
			**SDv**	±217.10	±193.96
			**CV [%]**	4.57	4.06
			**SDcv**	±1.97	±0.45
	**PC(18:1/18:0)**	**172.06**	**value [µg/mg]**	216.77	260.77
			**SDv**	±18.53	±17.59
			**CV [%]**	6.50	5.78
			**SDcv**	±2.33	±1.41
**(b)**	**LPE(14:0/0:0)**	**0.94**	**value [µg/mg]**	2.86	3.46
			**SDv**	±0.17	±0.26
			**CV [%]**	3.46	3.17
			**SDcv**	±1.74	±0.29
	**LPE(16:0/0:0)**	**33.56**	**value [µg/mg]**	10.08	11.97
			**SDv**	±1.08	±0.53
			**CV [%]**	4.35	4.07
			**SDcv**	±2.30	±0.62
	**LPE(18:0/0:0)**	**143.74**	**value [µg/mg]**	70.94	85.03
			**SDv**	±4.44	±4.57
			**CV [%]**	2.55	2.87
			**SDcv**	±1.48	±1.07

	PE(14:0/14:0)	**0.87**	**value [µg/mg]**	0.39	0.46
			**SDv**	±0.03	±0.02
			**CV [%]**	4.67	6.63
			**SDcv**	±2.15	±4.06
	PE(16:0/16:0)	**3.35**	**value [µg/mg]**	0.98	1.18
			**SDv**	±0.08	±0.06
			**CV [%]**	2.59	2.64
			**SDcv**	±0.83	±0.32
	PE(18:0/18:0)	**10.65**	**value [µg/mg]**	18.07	21.69
			**SDv**	±1.77	±1.56
			**CV [%]**	5.04	4.42
			**SDcv**	±2.90	±2.27
	PE(18:1(Z)/18:1(Z))	**12.53**	**value [µg/mg]**	19.37	23.33
			**SDv**	±1.65	±2.01
			**CV [%]**	6.25	5.56
			**SDcv**	±2.97	±2.96
	PE(18:1(E)/18:1(E))	**7.85**	**value [µg/mg]**	2.93	3.52
			**SDv**	±0.25	±0.25
			**CV [%]**	3.96	3.52
			**SDcv**	±2.06	±0.29
	PE(16:0/18:1)	**26.04**	**value [µg/mg]**	27.37	32.80
			**SDv**	±2.10	±1.81
			**CV [%]**	4.42	3.93
			**SDcv**	±1.86	±0.32
	PE(18:0/18:1)	**73.95**	**value [µg/mg]**	57.08	68.37
			**SDv**	±3.95	±3.45
			**CV [%]**	2.40	2.13
			**SDcv**	±1.02	±0.28
	PE(18:0/20:4)	**124.80**	**value [µg/mg]**	8.54	10.14
			**SDv**	±0.64	±0.33
			**CV [%]**	4.83	4.29
			**SDcv**	±3.55	±1.00
**(c)**	LPG(14:0/0:0)	**0.97**	**value [µg/mg]**	0.030	0.030
			**SDv**	±0.002	±0.002
			**CV [%]**	3.89	3.46
			**SDcv**	±1.93	±0.58
	LPG(16:0/0:0)	**10.72**	**value [µg/mg]**	3.94	4.74
			**SDv**	±0.32	±0.37
			**CV [%]**	2.14	1.90
			**SDcv**	±1.04	±0.54
	LPG(18:0/0:0)	**2.44**	**value [µg/mg]**	0.47	0.57
			**SDv**	±0.04	±0.03
			**CV [%]**	2.29	2.04
			**SDcv**	±1.23	±0.49
	PG(14:0/14:0)	**0.89**	**value [µg/mg]**	0.53	0.64
			**SDv**	±0.04	±0.04

			**CV [%]**	3.62	0.64
			**SDcv**	±1.86	±0.04
	**PG(16:0/16:0)**	**1.43**	**value [µg/mg]**	0.45	0.54
			**SDv**	±0.04	±0.03
			**CV [%]**	4.95	4.86
			**SDcv**	±2.65	±0.40
	**PG(18:0/18:0)**	**2.00**	**value [µg/mg]**	0.45	0.54
			**SDv**	±0.03	±0.03
			**CV [%]**	2.93	2.93
			**SDcv**	±2.00	±0.68
	**PG(18:1(Z)/18:1(Z))**	**2.50**	**value [µg/mg]**	5.67	6.80
			**SDv**	±0.39	±0.46
			**CV [%]**	5.42	4.82
			**SDcv**	±1.66	±0.91
	**PG(18:1(E)/18:1(E))**	**1.57**	**value [µg/mg]**	1.46	1.75
			**SDv**	±0.11	±0.10
			**CV [%]**	2.15	1.91
			**SDcv**	±0.80	±0.46
	**PG(18:2/18:2)**	**1.63**	**value [µg/mg]**	0.75	0.90
			**SDv**	±0.06	±0.05
			**CV [%]**	1.82	1.62
			**SDcv**	±0.61	±0.14
	**PG(18:0/18:1)**	**2.54**	**value [µg/mg]**	1.61	1.92
			**SDv**	±0.16	±0.08
			**CV [%]**	5.41	4.81
			**SDcv**	±2.33	±0.72
	**PG(18:0/22:6)**	**135.03**	**value [µg/mg]**	34.60	41.60
			**SD**	±2.75	±2.88
			**CV [%]**	4.59	4.08
			**SD**	±1.25	±0.59
**(d)**	**LPS(18:1/0:0)**	**6.97**	**value [µg/mg]**	4.00	4.78
			**SDv**	±0.31	±0.21
			**CV [%]**	3.18	2.83
			**SDcv**	±1.85	±0.42
	**PS(14:0/14:0)**	**1.44**	**value [µg/mg]**	0.68	0.81
			**SDv**	±0.06	±0.03
			**CV [%]**	3.13	2.78
			**SDcv**	±0.91	±0.28
	**PS(16:0/16:0)**	**1.49**	**value [µg/mg]**	0.12	0.15
			**SDv**	±0.009	±0.01
			**CV [%]**	2.09	1.86
			**SDcv**	±1.39	±0.78
	**PS(18:1(Z)/18:1(Z))**	**8.30**	**value [µg/mg]**	6.12	7.34
			**SDv**	±0.48	±0.42
			**CV [%]**	2.45	2.18
			**SDcv**	±1.28	±0.71

**PS(16:0/18:1)**	**5.64**	**value [µg/mg]**	4.74	5.67
		**SD$_v$**	±0.39	±0.28
		**CV [%]**	4.22	3.75
		**SD$_{CV}$**	±1.22	±0.73
**PS(18:0/18:1)**	**50.99**	**value [µg/mg]**	52.01	62.44
		**SD$_v$**	±3.82	±3.92
		**CV [%]**	2.50	2.22
		**SD$_{CV}$**	±1.06	±0.83
**PS(18:0/18:2)**	**7.17**	**value [µg/mg]**	6.56	7.86
		**SD$_v$**	±0.50	±0.44
		**CV [%]**	1.78	1.58
		**SD$_{CV}$**	±0.35	±0.33

Table S7. Table of the absolute concentrations [µg/mg], variance coefficients [%] and their standard deviation SD of the mouse cohort study separated into phenotypic groups (NT = non-tumor, T = tumor) including the significance level (Wilcoxon rank-sum test, 0.01<p<0.05: *; 0.001<p<0.01: **; 0.001<p<0.0001: ***; p<0.0001: ****). Analytes classes (a) PC - Phosphatidylcholine, (b) PE - Phosphatidylethanolamine, (c) PG - Phosphatidylglycerol, (d) PS - Phosphatidylserine.

(a) PC

	**LPC(14:0/0:0)**					**LPC(16:0/0:0)**					**LPC(18:0/0:0)**				
	**v [µg/mg]**	**SD**	**CV [%]**	**SD**	**p**	**v [µg/mg]**	**SD**	**CV [%]**	**SD**	**p**	**v [µg/mg]**	**SD**	**CV [%]**	**SD**	**p**
**NT**	0.82	±0.64	3.5	±3.29	0.35 / ns	0.07	±0.09	6.43	±4.60	0.012 / *	0.76	±0.87	6.43	±4.60	0.0015 / **
**T**	0.52	±0.29	5	±3.44		0.02	±0.03	3.61	±4.95		0.86	±0.48	3.61	±4.95	
	**PC(14:0/14:0)**					**PC(16:0/16:0)**					**PC(18:0/18:0)**				
	**v [µg/mg]**	**SD**	**CV [%]**	**SD**	**p**	**v [µg/mg]**	**SD**	**CV [%]**	**SD**	**p**	**v [µg/mg]**	**SD**	**CV [%]**	**SD**	**p**
**NT**	0.07	±0.12	0.76	±1.89	0.024 / *	0.01	±0.02	2.16	±4.76	0.0010 / **	33.68	±20.07	4.87	±4.06	0.000016 / ****
**T**	-	-	-	-		0.001	±0.002	0.29	±0.91		30.47	±14.84	5.77	±3.92	
	**PC(18:1(Z)/18:1(Z))**					**PC(18:1(E)/18:1(E))**					**PC(18:2/18:2)**				
	**v [µg/mg]**	**SD**	**CV [%]**	**SD**	**p**	**v [µg/mg]**	**SD**	**CV [%]**	**SD**	**p**	**v [µg/mg]**	**SD**	**CV [%]**	**SD**	**p**
**NT**	2.77	±1.55	4.25	±3.09	0.0000012 / ****	66.75	±43.57	6.04	±3.16	0.00000023 / ****	1.41	±1.02	4.43	±2.86	0.035 / *
**T**	2.49	±1.39	4.73	±3.28		97.65	±39.46	4.31	±3.26		0.89	±0.40	5.54	±3.74	
	**PC(16:0/18:0)**					**PC(16:0/18:1)/PC(18:1/16:0)**					**PC(16:0/20:4)**				
	**v [µg/mg]**	**SD**	**CV [%]**	**SD**	**p**	**v [µg/mg]**	**SD**	**CV [%]**	**SD**	**p**	**v [µg/mg]**	**SD**	**CV [%]**	**SD**	**p**
**NT**	0.72	±1.91	7.54	±3.40	0.000058 / ****	8.52	±5.04	3.07	±2.89	0.000033 / ****	26.86	±16.45	4.15	±3.17	0.00030 / ***
**T**	0.48	±0.26	6.77	±4.26		8.46	±4.17	4.98	±3.27		30.85	±16.97	5.31	±3.29	
	**PC(16:0/22:6)**					**PC(18:0/16:0)**					**PC(18:0/18:1)**				
	**v [µg/mg]**	**SD**	**CV [%]**	**SD**	**p**	**v [µg/mg]**	**SD**	**CV [%]**	**SD**	**p**	**v [µg/mg]**	**SD**	**CV [%]**	**SD**	**p**
**NT**	18.28	±11.42	3.06#	±3.01	0.000030 / ****	48.77	±35.05	4.8	±2.88	0.000020 / ****	16.53	±12.07	5.81	±4.48	0.000018 / ****
**T**	27.86	±13.28	4.71	±3.58		93.9	±43.65	5.56	±3.89		33.3	±18.56	5.14	±4.09	
	**PC(18:0/20:4)**					**PC(18:0/22:6)**					**PC(18:1/18:0)**				
	**v [µg/mg]**	**SD**	**CV [%]**	**SD**	**p**	**v [µg/mg]**	**SD**	**CV [%]**	**SD**	**p**	**v [µg/mg]**	**SD**	**CV [%]**	**SD**	**p**

**NT**	25.94	±18.42	5.9	±3.66	0.0014 / **	9.6	±8.01	8.33	±4.39	0.0052 / **	28.86	±11.89	4.32	±2.97	0.000048 // ****
**T**	56.65	±35.19	5.47	±3.87		18.5	±14.67	5.11	±3.94		43.9	±22.29	8.08	±10.86	
	**(b) PE**														
	**LPE(14:0/0:0)**					**LPE(16:0/0:0)**					**LPE(18:0/0:0)**				
	**v [µg/mg]**	**SD**	**CV [%]**	**SD**	**p**	**v [µg/mg]**	**SD**	**CV [%]**	**SD**	**p**	**v [µg/mg]**	**SD**	**CV [%]**	**SD**	**p**
**NT**	0.01	±0.04	0.52	±1.72	0.077 / ns	2.33	±1.52	6.45	±3.90	0.0020 / **	1.67	±0.96	6.62	±3.35	0.000026 / ****
**T**	0.02	±0.03	1.97	±2.89		4.02	±3.05	5.17	±4.12		2.71	±1.92	7.51	±4.22	
	**PE(14:0/14:0)**					**PE(16:0/16:0)**					**PE(18:0/18:0)**				
	**v [µg/mg]**	**SD**	**CV [%]**	**SD**	**p**	**v [µg/mg]**	**SD**	**CV [%]**	**SD**	**p**	**v [µg/mg]**	**SD**	**CV [%]**	**SD**	**p**
**NT**	0.2	±0.11	6.61	±3.72	0.0036 / **	0.04	±0.07	3.39	±4.26	0.021 / *	1.08	±0.48	7.05	±3.86	0.00069 / ***
**T**	0.34	±0.27	5.52	±4.06		0.01	±0.01	2.47	±3.60		0.92	±0.55	5.48	±4.23	
	**PE(18:1(Z)/18:1(Z))**					**PE(18:1(E)/18:1(E))**					**PE(16:0/18:1)**				
	**v [µg/mg]**	**SD**	**CV [%]**	**SD**	**p**	**v [µg/mg]**	**SD**	**CV [%]**	**SD**	**p**	**v [µg/mg]**	**SD**	**CV [%]**	**SD**	**p**
**NT**	0.18	±0.25	7.08	±3.97	0.0000045 / ****	3.56	±2.33	5.26	±3.47	0.000017 / ****	0.54	±0.37	5.57	±3.59	0.000083 / ****
**T**	0.17	±0.08	5.92	±3.51		7.03	±4.03	4.77	±2.90		1.11	±0.70	5.93	±3.15	
	**PE(18:0/18:1)**					**PE(18:0/20:4)**									
	**v [µg/mg]**	**SD**	**CV [%]**	**SD**	**p**	**v [µg/mg]**	**SD**	**CV [%]**	**SD**	**p**					
**NT**	19.92	±13.36	4.92	±3.48	0.00031 / ***	0.03	±0.05	1.84	±3.26	0.00089 / ***					
**T**	37.37	±20.17	4.45	±3.50		0.003	±0.005	1.68	±3.28						
	**(c) PG**														
	**LPG(14:0/0:0)**					**LPG(16:0/0:0)**					**LPG(18:0/0:0)**				
	**v [µg/mg]**	**SD**	**CV [%]**	**SD**	**p**	**v [µg/mg]**	**SD**	**CV [%]**	**SD**	**p**	**v [µg/mg]**	**SD**	**CV [%]**	**SD**	**p**
**NT**	0.26	±0.20	5.81	±3.57	0.30 / ns	4.25	±2.98	5.93	±4.32	0.84 / ns	254.4	±189.49	5.47	±3.60	0.56 / ns
**T**	0.47	±0.36	6.82	±3.74		7.6	±3.68	2.71	±2.30		402.27	±209.77	6.25	±4.54	
	**PG(14:0/14:0)**					**PG(16:0/16:0)**					**PG(18:0/18:0)**				
	**v [µg/mg]**	**SD**	**CV [%]**	**SD**	**p**	**v [µg/mg]**	**SD**	**CV [%]**	**SD**	**p**	**v [µg/mg]**	**SD**	**CV [%]**	**SD**	**p**

**NT**	12.03	±6.26	3.63	±2.64	0.20 / ns	104.18	±69.68	6.87	±4.03	0.41 / ns	21.42	±15.11	5.01	±3.85	0.71 / ns
**T**	20.37	±12.17	5.86	±3.60		198.38	±93.29	6.86	±10.27		40.65	±23.79	3.8	±2.98	
	**PG(18:1(Z)/18:1(Z))**					**PG(18:1(E)/18:1(E))**					**PG(18:2/18:2)**				
	**v [µg/mg]**	**SD**	**CV [%]**	**SD**	**p**	**v [µg/mg]**	**SD**	**CV [%]**	**SD**	**p**	**v [µg/mg]**	**SD**	**CV [%]**	**SD**	**p**
**NT**	1.28	±0.97	5.29	±6.32	0.050 / *	2959.17	±2123.84	5.3	±7.09	0.014 / *	475.15	±330.4 1	7.52	±3.59	0.014 / *
**T**	1.77	±1.05	5.14	±3.84		3858.83	±1958.19	15.12	±13.53		832.92	±393.9 5	6.26	±4.13	
	**PG(18:0/18:1)**					**PG(18:0/22:6)**									
	**v [µg/mg]**	**SD**	**CV [%]**	**SD**	**p**	**v [µg/mg]**	**SD**	**CV [%]**	**SD**	**p**					
**NT**	16.78	±10.89	5.28	±6.01	0.70 / ns	64.08	±45.01	5.21	±3.81	0.33 / ns					
**T**	29.12	±15.58	6.28	±3.07		99.67	±53.39	6.84	±4.26						
	**(d) PS**														
	**LPS(18:1/0:0)**					**PS(14:0/14:0)**					**PS(16:0/16:0)**				
	**v [µg/mg]**	**SD**	**CV [%]**	**SD**	**p**	**v [µg/mg]**	**SD**	**CV [%]**	**SD**	**p**	**v [µg/mg]**	**SD**	**CV [%]**	**SD**	**p**
**NT**	0.51	±1.08	4.92	±4.64	0.0000065 / ****	1.74	±1.36	7.72	±4.00	0.0033 / **	39.48	±25.40	6.67	±6.17	0.011 / *
**T**	0.79	±0.50	5.68	±4.71		3.34	±2.21	5.32	±3.68		59.98	±33.22	6.27	±3.85	
	**PS(18:1(Z)/18:1(Z))**					**PS(16:0/18:1)**					**PS(18:0/18:1)**				
	**v [µg/mg]**	**SD**	**CV [%]**	**SD**	**p**	**v [µg/mg]**	**SD**	**CV [%]**	**SD**	**p**	**v [µg/mg]**	**SD**	**CV [%]**	**SD**	**p**
**NT**	62.19	±45.25	5.67	±3.79	0.00033 / ***	-	-	-	-	-	15.8	±11.36	6.87	±5.14	0.0033 / **
**T**	91.01	±46.13	6.42	±4.37		0.07	±0.17	-	-		29.55	±15.66	5.09	±4.12	
	**PS(18:0/18:2)**														
	**v [µg/mg]**	**SD**	**CV [%]**	**SD**	**p**										
**NT**	3.04	±2.10	2.41	±2.69	0.00000073 / ****										
**T**	6.17	±3.61	5.54	±3.39											

Figure S1. Graphical representations of absolute concentrations [µg/mg] of phosphatidylcholines in comparison of phenotypic differences of the tissue (NT = non-tumor, T = tumor), including the significance level (0.01<p<0.05: *; 0.001<p<0.01: **; 0.001<p<0.01: ***; p<0.0001: ****).

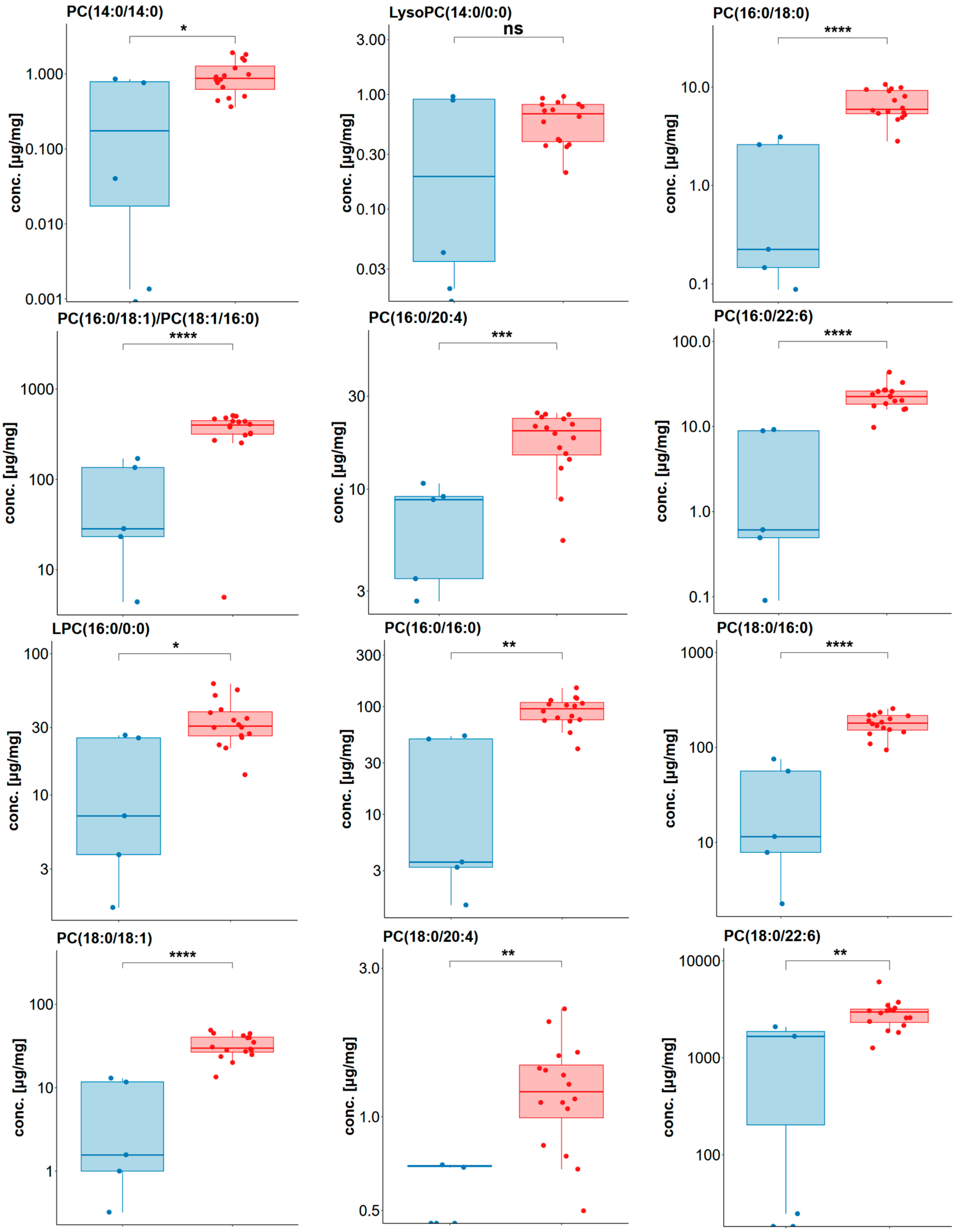

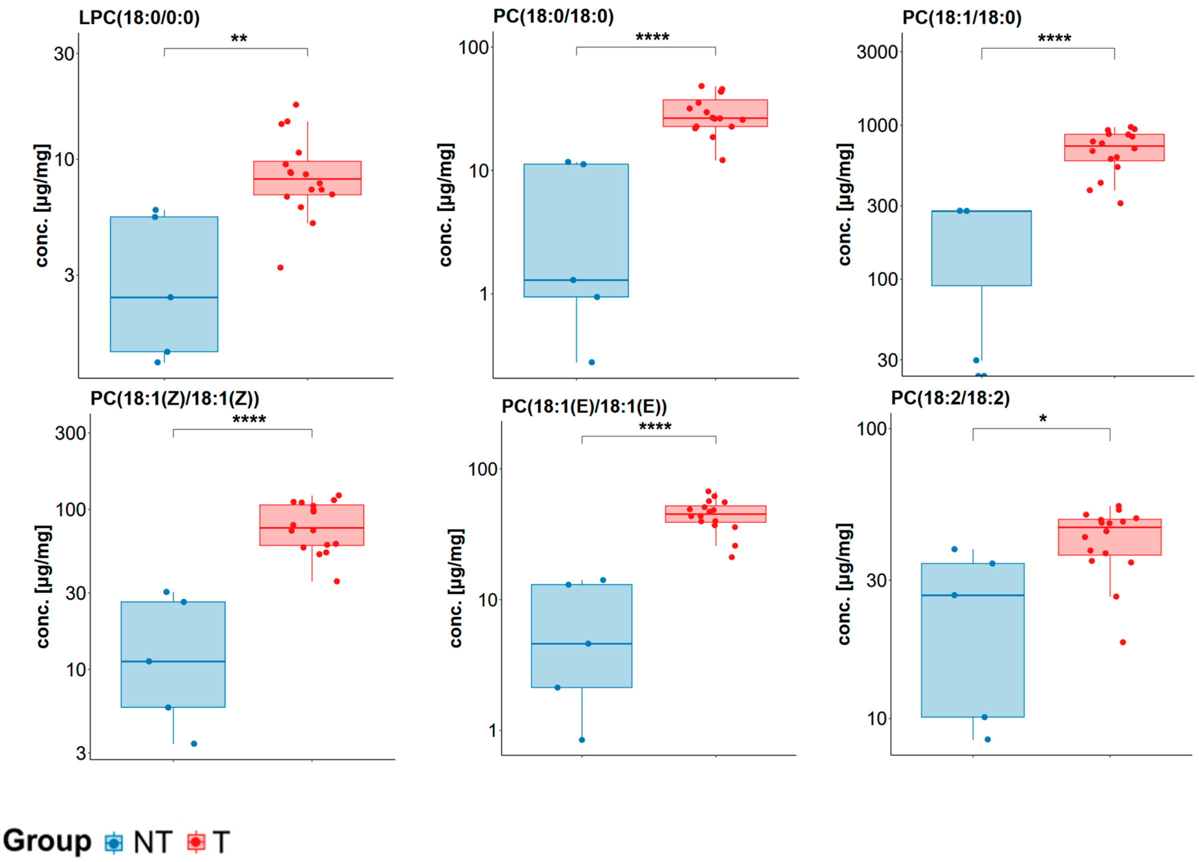
LPC(18:0/0:0)
**
30
10
3
conc. [µg/mg]
PC(18:0/18:0)
****
100
10
1
conc. [µg/mg]
PC(18:1/18:0)
****
3000
1000
300
100
30
conc. [µg/mg]
PC(18:1(Z)/18:1(Z))
****
300
100
30
10
3
conc. [µg/mg]
PC(18:1(E)/18:1(E))
****
100
10
1
conc. [µg/mg]
PC(18:2/18:2)
*
100
30
10
conc. [µg/mg]
Group NT T

Figure S2. Graphical representations of absolute concentrations [µg/mg] of phosphatidylethanolamines in comparison of phenotypic differences of the tissue (NT = non-tumor, T = tumor), including the significance level (0.01<p<0.05: *; 0.001<p<0.01: **; 0.001<p<0.01: ***; p<0.0001: ****). .

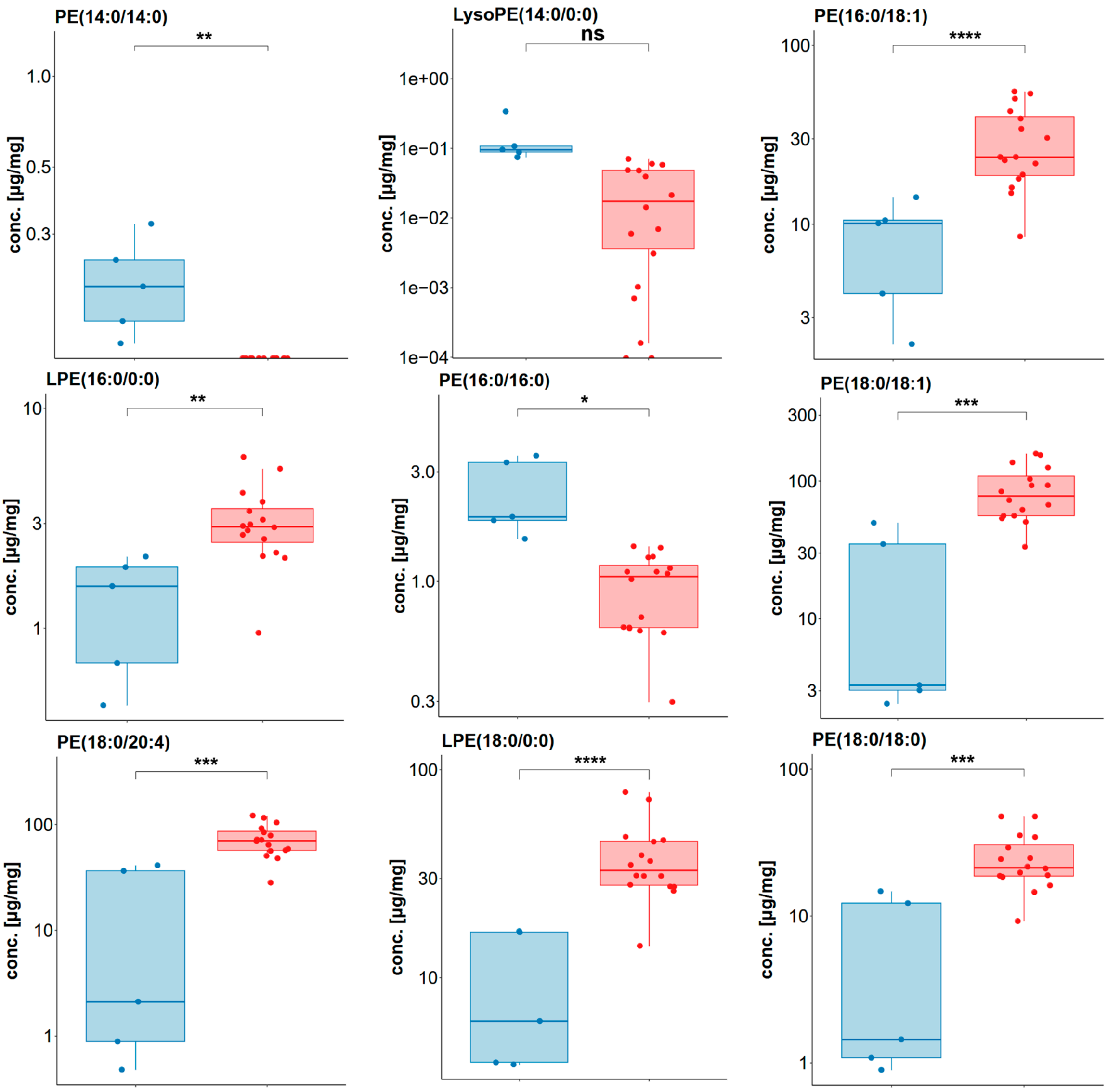

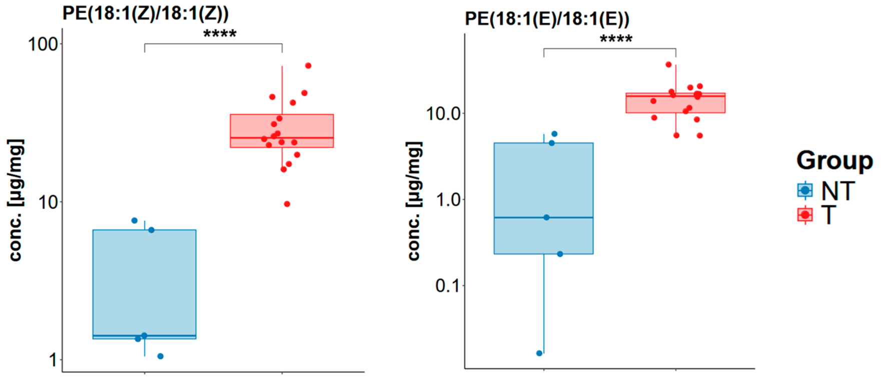

PE(18:1(Z)/18:1(Z))
****
100
10
1
conc. [µg/mg]
PE(18:1(E)/18:1(E))
****
10.0
1.0
0.1
conc. [µg/mg]
Group
NT
T

Figure S3. Graphical representations of absolute concentrations [µg/mg] of phosphatidylglycerols in comparison of phenotypic differences of the tissue (NT = non-tumor, T = tumor), including the significance level (0.01<p<0.05: *; 0.001<p<0.01: **; 0.001<p<0.01: ***; p<0.0001: ****).

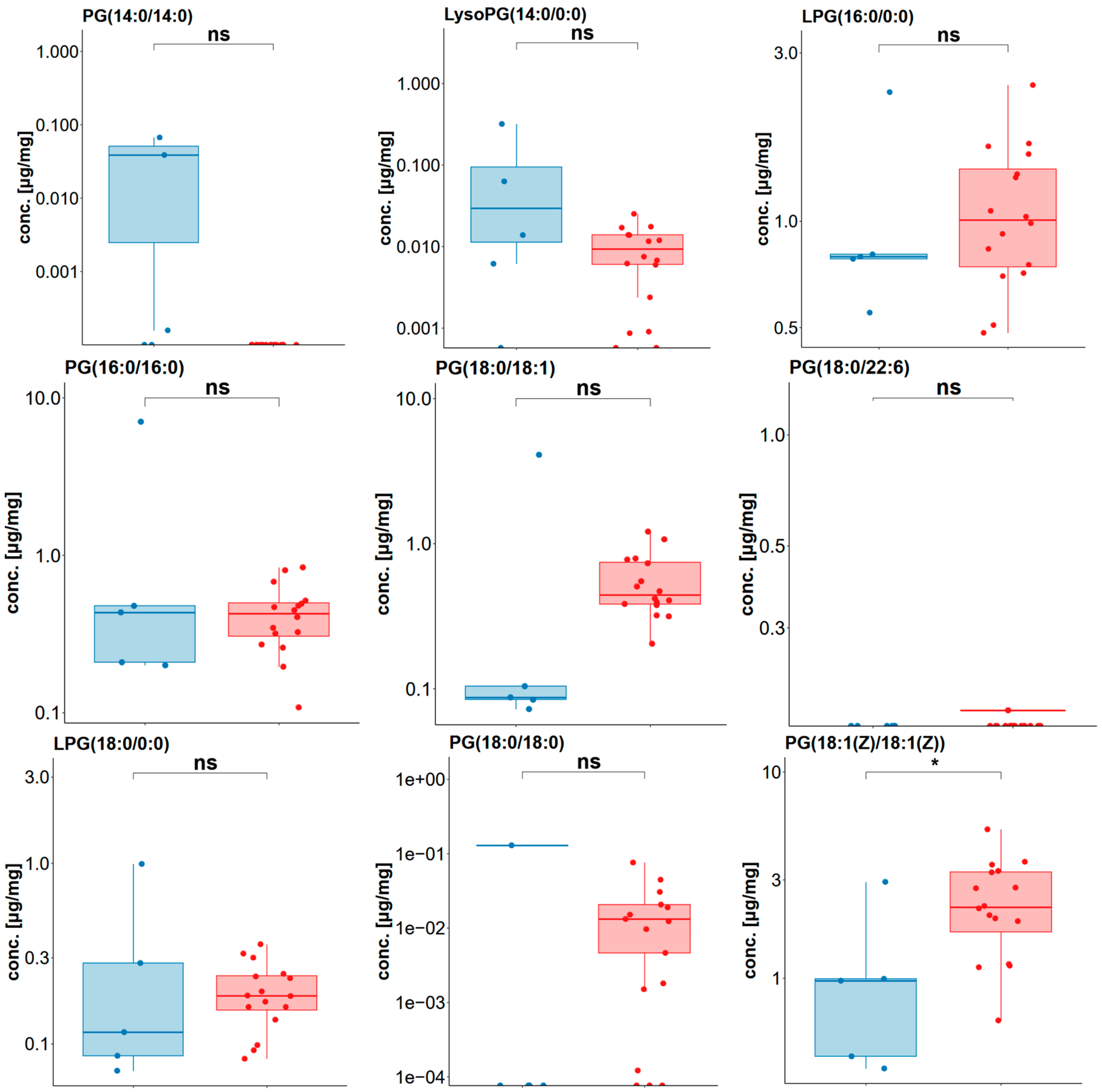

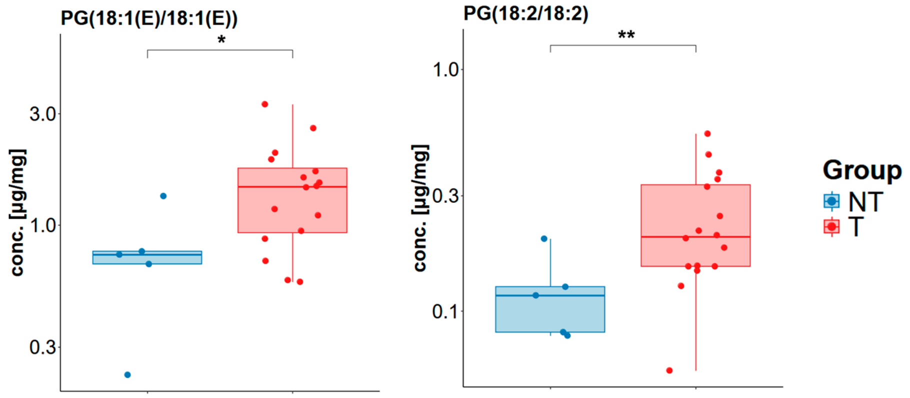
PG(18:1(E)/18:1(E))
*
3.0
1.0
0.3
conc. [µg/mg]
PG(18:2/18:2)
**
1.0
0.3
0.1
conc. [µg/mg]
Group
NT
T

Figure S4. Graphical representations of absolute concentrations [µg/mg] of phosphatidylserines in comparison of phenotypic differences of the tissue (NT = non-tumor, T = tumor), including the significance level (0.01<p<0.05: *; 0.001<p<0.01: **; 0.001<p<0.01: ***; p<0.0001: ****).

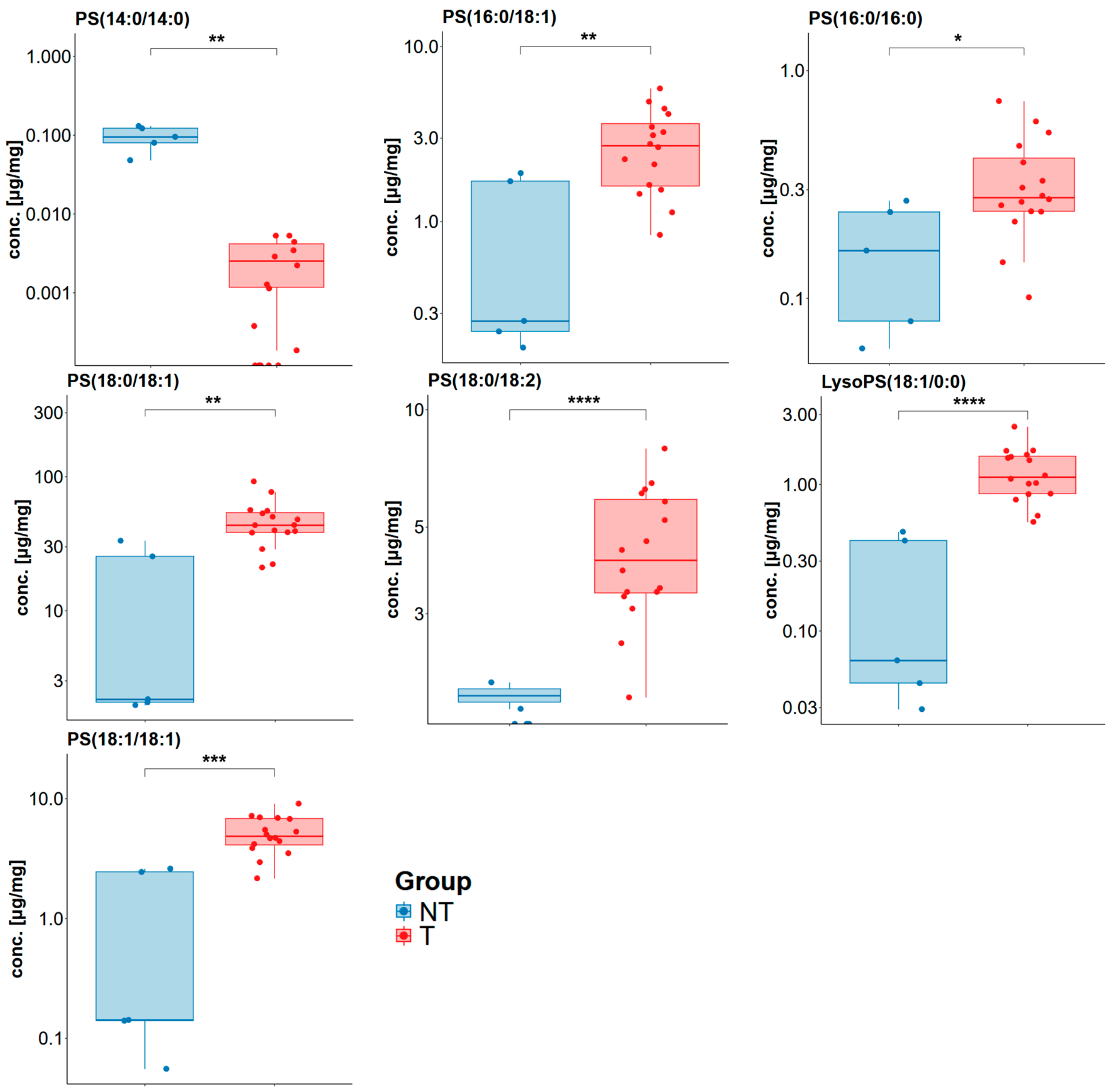

Mandel F-value [adapted from ref. [17]]

Residuals:

$$Residuum_i = y_i - \hat{y}_i$$

$y_i = meassured\ value$
$\hat{y}_i = calculated\ value$

Residual standard deviation s :

$$s_{lin}/s_{squred} = \sqrt{\frac{\sum(y_i - \hat{y}_i)^2}{n-p}}$$

$n = calibration\ points$
$p = number\ of\ model\ parameters$

- $linear: p = 2$
- $squared: p = 3$

Mandel-F-Value:

$$F_{Mandel} = \frac{s_{lin}^2 - s_{squared}^2}{s_{squared}^2} * (n-3)$$

$F_{Mandel} > F_{krit} : squared$
$F_{Mandel} \leq F_{krit} : linear$
